# Facile and scale-up syntheses of high-performance enzyme@meso-HOF biocatalysts†

**DOI:** 10.1039/d4sc04619f

**Published:** 2024-09-20

**Authors:** Zhengyi Di, Yu Qi, Xin-Xin Yu, Hai-Ruo Li, Meng-Xuan Zuo, Tian-Tian Ren, Cheng-Peng Li, Yanli Zhao

**Affiliations:** a College of Chemistry, Tianjin Key Laboratory of Structure and Performance for Functional Molecules, Tianjin Normal University Tianjin 300387 China hxxylcp@tjnu.edu.cn; b School of Chemistry, Chemical Engineering and Biotechnology, Nanyang Technological University 21 Nanyang Link 637371 Singapore zhaoyanli@ntu.edu.sg

## Abstract

Facile immobilization is essential for the wide application of enzymes in large-scale catalytic processes. However, exploration of suitable enzyme supports poses an unmet challenge, particularly in the context of scale-up biocatalyst fabrication. In this study, we present facile and scale-up syntheses of high-performance enzyme biocatalysts *via in situ* encapsulation of cytochrome c (Cyt-c) as mono-enzyme and glucose oxidase (GOx)-horseradish peroxidase (HRP) as dual-enzyme cascade (GOx&HRP) systems, respectively, into a stable mesoporous hydrogen-bonded organic framework (meso-HOF) matrix. *In situ* encapsulation reactions occur under ambient conditions, and facilitate scale up (∼3 g per reaction) of enzyme@meso-HOF within a very short period (5–10 min). The resultant biocatalysts not only exhibit high enzyme loading (37.9 wt% for mono-enzyme and 22.8 wt% for dual-enzyme) with minimal leaching, but also demonstrate high catalytic activity, superior reusability, and durability. This study represents an example of scale-up fabrication of enzyme@meso-HOF biocatalysts on the gram level and highlights superior meso-HOFs as suitable host matrices for biomolecular entities.

## Abstract 2

Facile and scale-up fabrication of enzyme-loaded mesoporous hydrogen-bonded organic frameworks as efficient biocatalysts is achieved.

## Graphical abstract

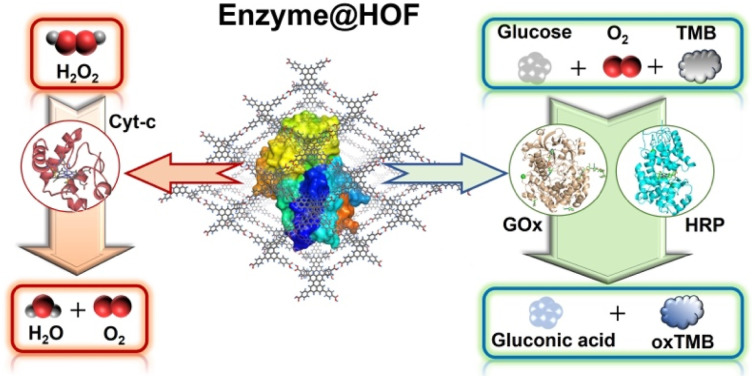

## Introduction

Enzymes, as biological catalysts, play critical roles in nearly all biochemical processes, exhibiting high specificity and activity to accelerate chemical reactions at ambient pressure and temperature.^1–3^ These attributes position enzymes as the “core chip” in various scientific and industrial domains, including biotechnology,^4^ the food industry,^5^ drug delivery,^6^ medical diagnostics,^7^ the pharmaceutical industry,^8,9^ and biofuel manufacturing.^10,11^ The global enzyme market is projected to reach $13.2 billion by 2027, with a compound annual growth rate of 8%.^12^ However, the use of free enzymes presents challenges such as high cost, poor stability, and limited reusability.^13^ These obstacles impede the scaling up of free enzymes from the laboratory or pilot-scale to industrial production levels.^14^ Immobilizing enzymes into/onto porous solid supports is considered an effective solution to address these challenges. Despite efforts to develop high-performance enzyme immobilization systems by adjusting matrices (*e.g.*, organic polymers or inorganic metal oxides)^15–18^ and immobilization strategies (*e.g.*, adsorption, covalent bonding, or crosslinking),^19–21^ current systems still face limitations, including difficulty in encapsulating large enzymes, inefficient substrate/product transfer due to nonporous carriers, and enzyme denaturation and leaching.^22^ Therefore, expedited fabrication of high-performance immobilized biocatalysts on a large scale remains a pressing need.^23^ In contrast to surface immobilization methods, the *in situ* enzyme encapsulation strategy offers a versatile and effective approach by facilitating simultaneous self-assembly of enzymes and building units of the host matrix.^24^ This approach aims to protect or stabilize enzymes within a specific environment, preserving their activity and functionality.^25^ Notably, this strategy enables facile encapsulation of enzymes under mild conditions, such as an aqueous phase, reasonable pH, and room temperature.^12^ Furthermore, to enhance the contact rate between the reaction substrate and the loaded enzyme, the host matrix should possess high porosity and ordered open channels.^26^

To date, crystalline porous solids such as metal–organic frameworks (MOFs),^27–29^ covalent-organic frameworks (COFs),^30,31^ and hydrogen-bonded organic frameworks (HOFs)^32,33^ have emerged as effective carriers for addressing the challenges of *in situ* enzyme encapsulation.^34^ These materials offer tunable structures with easy functionalization and adjustable pore sizes within the mesoporous range.^35^ Compared to MOFs and COFs that are constructed from metal coordination and covalent bonding interactions, HOFs are assembled through weak hydrogen-bonding contacts aided by rich π–π stacking interactions.^36–38^ This approach endows HOFs with highly dynamic and flexible structures.^39–41^ The metal-free nature of HOFs provides better biocompatibility and lower toxicity, making them highly desirable for biocatalyst and biomedicine applications. Furthermore, HOFs can self-assemble in solutions at ambient temperature, allowing enzymes to be immobilized without degradation.^42^ Despite the reported success of some HOF-based bio-immobilization matrices, there is still a pending trade-off between increasing the open aperture size and improving the stability of HOFs.^43,44^ Specifically, achieving a combination of large pore size and high stability within a single HOF material remains an unmet challenge.^45–47^

The ligand modification and extension approaches have been widely used in the design of MOFs and COFs to fabricate mesoporous solids.^48–53^ This sophisticated approach involves extending ligands or monomers by introducing enlarged linkers between reactive groups, albeit with the inherent risk of network interpenetration.^54,55^ Notably, this strategy has also been applied to design a series of stable two-dimensional (2D) HOFs, known as HOF-10*x* (*x* = 0, 1, 2).^56^ By incorporating benzene and naphthalene spacers between perylene and carboxyl groups in the HOF-10*x* monomers, the channel sizes exhibit a systematic increase from HOF-100 to HOF-101 and HOF-102. However, increasing the length of HOF monomers may lead to undesirable network interpenetration or layer staggering arrangements, resulting in decreased pore size and compromised stability.^39,57,58^ With these in mind, we propose that HOF monomers with bulky fused aromatic rings could effectively address the trade-off between porosity and stability within a single HOF. Specifically, expanding the backbone of the conjugated system in HOFs is anticipated to enhance pore size while promoting more stable π–π interactions between the eclipsed 2D layers simultaneously.

**Fig. 1:**
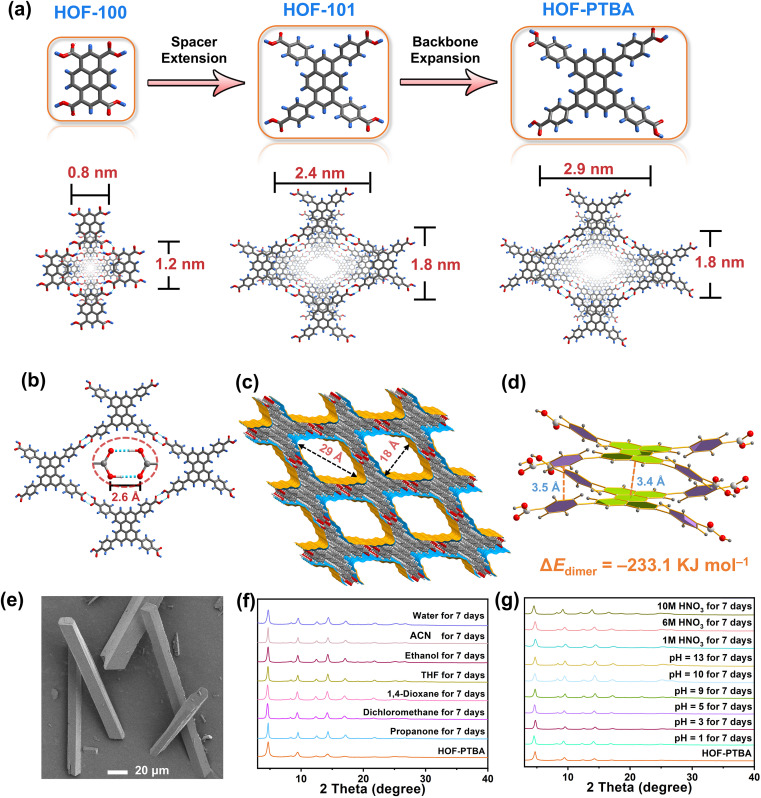
(a) Fabrication strategy of HOF-PTBA by the backbone expansion approach. (b) 2D H-bonding layer in HOF-PTBA, highlighting the O–H⋯O bonds. (c) 1D channels (18 × 29 Å) in HOF-PTBA. (d) Face-to-face π–π interactions (interlayer distance 3.4 and 3.5 Å) in HOF-PTBA. (e) SEM image of HOF-PTBA. (f) PXRD patterns for structural stability of HOF-PTBA under various pH conditions for 7 days. (g) PXRD patterns of HOF-PTBA after immersing in different solvents for 7 days.

To validate this hypothesis, we are motivated by using a bulky π-conjugation system of perylene as the backbone of HOF monomers to construct ultrastable mesoporous HOF-PTBA (Fig. 1a), serving as a host matrix for *in situ* encapsulation of enzymes. HOF-PTBA can be readily scaled up for preparation under mild conditions (aqueous solution and room temperature). It features a one-dimensional (1D) square channel measuring 18 × 29 Å and remains stable in various environments, including water, organic solvent, 10 M HNO_3_ and 0.1 M NaOH for at least 7 days. These features enable us to develop an enzyme immobilization scaffold for fabricating enzyme@meso-HOF biocatalysts. To assess the universality of this approach, we select cytochrome c (Cyt c) as mono-enzyme and glucose oxidase (GOx)-horseradish peroxidase (HRP) as dual-enzyme cascade (GOx&HRP) systems, and confine them within HOF-PTBA mesoporous environments. Notably, *in situ* encapsulation reactions occur under ambient conditions, and facilitate scale up (∼3 g per reaction) of enzyme@HOF within a very short period (5–10 minutes). The resulting enzyme@HOF materials demonstrate high enzyme loading with minimal leaching, high catalytic activity, and superior reusability and durability. This study represents a pioneer example for the scale-up fabrication of enzyme@HOF biocatalysts, and highlights meso-HOFs as host matrices for biomolecular entities.

## Results and discussion

To address the trade-off effect between stability and porosity of crystalline frameworks, we design and fabricate a mesoporous HOF with a pore size of 18 × 29 Å by employing a novel backbone extension strategy (Fig. 1a). This strategy offers several advantages: firstly, the introduction of matrices with larger π-conjugation creates HOFs with higher porosity. Secondly, bulky aromatic building units of HOFs enhance the interactions between the H-bonded 2D layers. Thirdly, the ordered and porous channels in HOFs would increase the transport rate of substrates, thereby improving the efficiency of enzyme catalysis. Additionally, the crystal structure can be obtained through single-crystal X-ray diffraction measurements, which facilitate the determination of the spatial positions of the atoms and provides a theoretical basis for the design and synthesis of more stable HOF structures.

The 1,3,6,8-tetrakis(benzoic acid)perylene (H_4_PTBA) organic building block was obtained from a 3-step synthesis, as illustrated in Fig. S1,† and confirmed by ^1^H NMR (Fig. S2–S4†). The molecular structures of HOF-100, HOF-101 and HOF-PTBA are shown in Fig. S5.† High-quality single crystals of HOF-PTBA were synthesized by slow vapor diffusion from the DMF-MeOH medium at room temperature (Fig. S6†). Single-crystal X-ray diffraction studies revealed that HOF-PTBA crystallizes in the monoclinic space group *C*2/*m* (Table S1†). The asymmetric unit of HOF-PTBA contains one-quarter H_4_PTBA molecule, which remains undeprotonated (Fig. S7†). Each H_4_PTBA molecule interacts with four neighbouring molecules through eight O–H⋯O hydrogen bonds, extending into a 2D layer. The O–H⋯O distance measures 2.6 Å (Fig. 1b and S8†), falling within the strong hydrogen bond range. Each 2D square layer interacts with adjacent layers through face-to-face π–π stacking interactions (interlayer distance 3.4 Å) to form a 1D square channel of 18 × 29 Å (Fig. 1c and S9–S11†). The permanent porosity of HOF-PTBA was disclosed by N_2_ sorption measurement at 77 K, which shows that HOF-PTBA possesses the typical type IV sorption profile, indicating its mesoporous nature (Fig. S12†). The pore size distribution profiles derived from these isotherms were centered at about 2.3 nm (Fig. S13†).

As depicted in Fig. 1d and S14,† the bulky π-conjugated perylene groups in HOF-PTBA are stacked in an AA arrangement, significantly increasing the aromatic stacking energy. The energy for the HOF-PTBA dimer is −233.1 kJ mol^−1^, which is much higher than the hydrogen bonding energy of −46.6 kJ mol^−1^, demonstrating the vital role of π–π stacking in structural stability. In comparison, the energy for the PFC-1 dimer is −199.8 kJ mol^−1^, with a hydrogen bonding energy of −47.1 kJ mol^−1^. Consequently, HOF-PTBA exhibits stronger π-conjugated interactions and thus a more stable structure than PFC-1. Meanwhile, the peaks of PTBA at 1418 and 1290 cm^−1^ were found to be red-shifted to 1409 and 1285 cm^−1^ in HOF-PTBA from the FT-IR spectra, indicating the presence of π–π stacking interactions (Fig. S15†).^59^

The as-synthesized (Fig. 1e) and scale-up products of HOF-PTBA are nearly identical, as determined by powder X-ray diffraction (PXRD) (Fig. S16†). Even after being soaked in 1 M, 6 M and 10 M HNO_3_ aqueous solutions for 7 days, HOF-PTBA maintains its crystallinity well. Remarkably, HOF-PTBA retains its crystallinity even under strong alkaline conditions at pH = 13 for 7 days, a rarity for carboxyl-HOFs (Fig. 1f). For comparison, PFC-1 is only stable for 14 hours at pH = 13, providing the basis for HOF-PTBA to be used in more harsh environments. Furthermore, HOF-PTBA remains intact in different solvents for 7 days, which include water, acetonitrile (ACN), ethanol, tetrahydrofuran (THF), 1,4-dioxane, dichloromethane, and propanone (Fig. 1g). Even after 545 days of immersion in various solvents, HOF-PTBA still maintained good crystallinity (Fig. S17†). The thermal stability of HOF-PTBA was confirmed by thermogravimetric analysis (TGA), showing that the framework remained unchanged up to 440 °C (Fig. S18†). Therefore, the excellent solvent stability, acid and alkali resistance, and thermal stability of HOF-PTBA make it a potential support for enzyme immobilization.

**Fig. 2:**
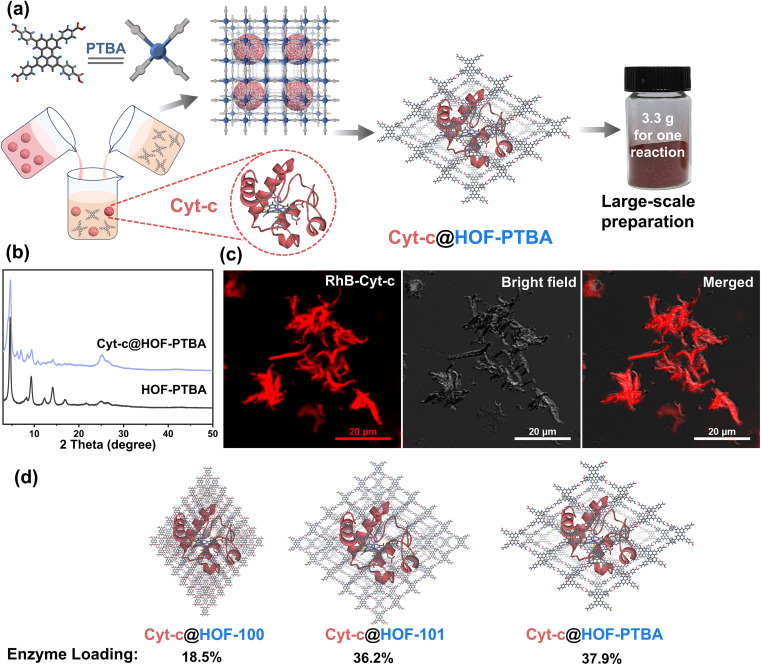
(a) Facile synthesis of Cyt-c@HOF-PTBA at room temperature and a digital photograph based on a scale-up synthesis (3.3 g for one reaction). (b) PXRD patterns of Cyt-c@HOF-PTBA and HOF-PTBA. (c) CLSM images of Cyt-c@HOF-PTBA (Cyt-c was labelled by using a red dye). (d) Calculated Cyt-c loadings by different HOFs supported by the ICP-MS measurements.

To demonstrate the feasibility of HOF-PTBA for enzyme loading, we chose Cyt-c as a representative biocatalyst. Encapsulation of Cyt-c into HOF-PTBA was achieved through a biomimetic mineralization process. The enzyme solution was added to the solution containing the H_4_PTBA monomer by a simple one-pot method (Fig. 2a). After stirring for about 5 minutes at room temperature, the biocomposite (named Cyt-c@HOF-PTBA) was isolated on the gram level (∼3.3 g per reaction). The weight loss of Cyt-c@HOF-PTBA in the range of 290–440 °C in the TG curves was caused by the pyrolysis of the incorporated Cyt-c (Fig. S18†). As depicted in Fig. 2b, the framework of HOF-PTBA remains unchanged after enzyme immobilization, as revealed by PXRD. The distribution of Cyt-c in Cyt-c@HOF-PTBA was characterized by confocal laser scanning microscopy (CLSM) experiments, wherein Cyt-c was labelled with rhodamine B (RhB), a red fluorescent dye.^26^ The red fluorescence completely covered the material (Fig. 2c), suggesting uniform spatial distribution of Cyt-c. According to the results from CLSM, the enzymes were evenly distributed throughout the entire structure of the HOF, rather than located inside the pores.^60–62^ The standard Bradford assay (Fig. S19†) indicated a Cyt-c content of 40.8 wt% in the nanosystem. Such a high enzyme loading was also confirmed by inductively coupled plasma-mass spectra (ICP-MS) (Fig. S20†), wherein Cyt-c was calculated on average to be 37.9 wt% in Cyt-c@HOF-PTBA (Table S2†). This value exceeded that of HOF-100 (18.5 wt%) and HOF-101 (36.2 wt%) (Fig. 2d). These results clearly confirmed the successful encapsulation of Cyt-c within the pores of HOF-PTBA to form the Cyt-c@HOF-PTBA biocomposite.

**Fig. 3:**
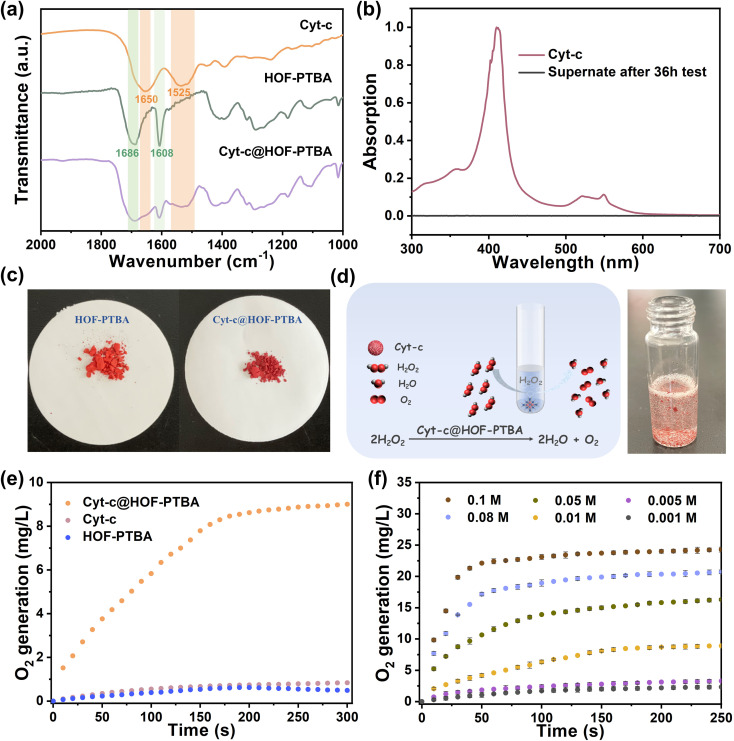
(a) FT-IR spectra of HOF-PTBA, Cyt-c@HOF-PTBA, and Cyt-c. (b) UV-vis spectra of the collected supernatants after the biomimetic mineralization process. (c) Color comparison between HOF-PTBA and Cyt-c@HOF-PTBA. (d) Catalytic reaction and digital photograph showing O_2_ bubble generation using the Cyt-c@HOF-PTBA catalyst. (e) Capacity of O_2_ generation *via* H_2_O_2_ decomposition by Cyt-c@HOF-PTBA, Cyt-c and HOF-PTBA. (f) H_2_O_2_-dependent catalytic kinetic curves of the Cyt-c@HOF-PTBA catalyst. All tests were carried out in Tris buffer (pH 7.5, 50 mM). Cyt c dosage was kept at 0.1 mg mL^−1^.

To verify the stability of the encapsulated material, we soaked 1.5 mg of Cyt-c@HOF-PTBA in 5 mL of aqueous solution for 36 h. As shown in Fig. 3a, the FT-IR spectrum of Cyt-c@HOF-PTBA revealed the presence of amide I (1650 cm^−1^) and amide II (1525 cm^−1^) bands of Cyt-c,^59^ indicating that Cyt-c remained encapsulated in the HOF. To accurately determine the interfacial interactions between Cyt-c and the host HOF matrix, solid-state NMR (ssNMR) spectroscopy was employed.^63,64^ As a result, the ssNMR spectra of Cyt-c@HOF-PTBA were obviously different from those of the free enzyme and the physical mixture of the enzyme and HOF (Fig. S21†). In the ^1^H and ^13^C ssNMR spectra of HOF-PTBA, the chemical shifts of the carboxyl group were in the ranges of 19–20 ppm and 164–165 ppm respectively, which were found to be shifted in Cyt-c@HOF-PTBA. Thus, additional interactions between Cyt-c and HOF-PTBA facilitate enzyme immobilization.^59^ Moreover, no absorption peak was observed in the UV-vis spectrum of the supernatant after soaking in aqueous solution for 36 h , showing no obvious leaching of Cyt-c after treatment (Fig. 3b). Notably, the color of the materials darkened after immobilization, attributed to Cyt-c being encapsulated within the framework of HOF-PTBA (Fig. 3c).

The coordination environments of heme in both free Cyt-c and Cyt-c@HOF-PTBA were examined using electron paramagnetic resonance (EPR) spectroscopy. The results revealed that both forms contained high-spin ferric heme with g-values of 5.97 and 5.68 (Fig. S22†). However, no low-spin ferric heme signal (*g* = 2.93 and 2.29) was detected in Cyt-c@HOF-PTBA, as observed in previous studies.^65,66^ These results indicate that encapsulation within HOF-PTBA alters the conformation of Cyt-c, leading to the observed catalase-like biocatalytic properties in Cyt-c@HOF-PTBA.

To assess the feasibility of enzyme encapsulation, we evaluated the biocatalytic performance of Cyt-c@HOF-PTBA (Fig. 3d). Interestingly, Cyt-c@HOF-PTBA exhibited catalase-like bioactivity, decomposing H_2_O_2_ into O_2_ and H_2_O (Fig. 3e and S23†).^59^Fig. 3f illustrates that the catalytic activity of Cyt-c@HOF-PTBA was dependent on substrate concentration (0.001–0.1 M), with higher concentrations yielding greater activity. Specifically, at an H_2_O_2_ concentration of 0.1 M, the oxygen yield in the presence of Cyt-c@HOF-PTBA reached 22.1 mg L^−1^ in 50 seconds, representing a 1.95-fold increase in the catalytic rate compared to that of Cyt-c@HOF-101 (Table S3†).^59^ To further evaluate the catalytic activities of Cyt-c encapsulated by HOF-PTBA and HOF-101, the Michaelis–Menten model was employed (see the ESI† for details). As a result, the Michaelis constant *K*_m_ of Cyt-c@HOF-PTBA is 29.7 mM s^−1^, being lower than that of Cyt-c@HOF-101 (32.3 mM s^−1^), which indicates that the binding interactions between the enzyme and the substrate in Cyt-c@HOF-PTBA are stronger (Fig. S24 and Table S4†). These findings indicate that a larger pore structure enhances the catalytic efficiency of the loaded enzyme.

**Fig. 4:**
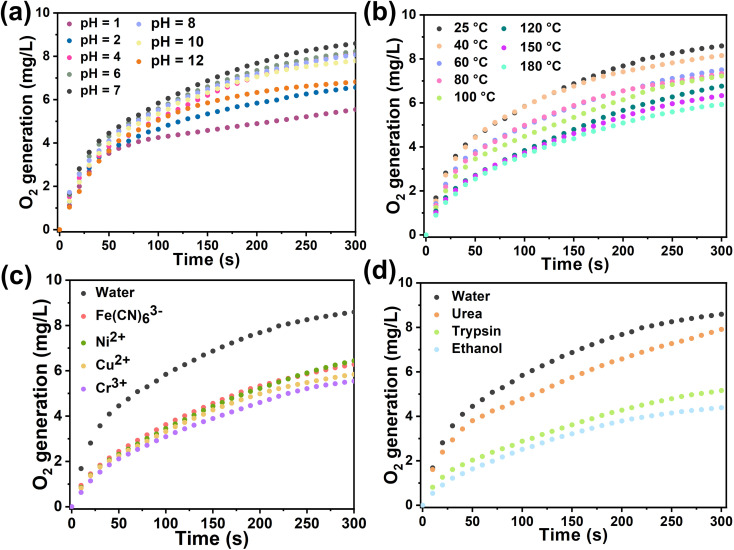
Catalytic kinetics of Cyt-c@HOF-PTBA after treatment at non-physiological pH (a), by heating for 30 min (b), and using heavy metal ions (c) and denaturing reagents and organic solvents (d).

In general, biological enzyme catalysts are costly and difficult to recover, and their catalytic activity is sensitive to external conditions such as temperature and pH (Fig. S25†). Immobilizing enzymes within HOF pores may mitigate these issues and advance enzyme development in industry. We assessed the catalytic kinetics of the composites by subjecting Cyt-c@HOF-PTBA to various pH solutions for 30 minutes at room temperature. As a result, the biocomposite maintained efficient H_2_O_2_ catalytic activity over a wide pH range under acidic (pH = 1) to basic (pH = 12) conditions (Fig. 4a). At pH = 2, Cyt-c@HOF-PTBA exhibited 1.61 times higher catalytic activity than Cyt-c@HOF-101. Even at pH = 12, Cyt-c@HOF-PTBA still retained 80% catalytic activity, owing to the extensive π-conjugation system of HOF-PTBA providing effective protection for Cyt-c under extreme pH conditions. Temperature significantly impacts enzyme catalysis activity, often leading to deactivation at high temperatures, thus requiring storage at low temperatures. We subjected Cyt-c@HOF-PTBA to temperatures ranging from 25 to 180 °C for 30 minutes (Fig. 4b), and evaluated its H_2_O_2_ catalytic ability. Catalytic activity remained relatively stable up to 40 °C (activity: 95%). Even at 100 °C, catalysis activity was sustained at 87%. Notably, at 180 °C, catalytic activity was still considered high (activity: 69%), enabling enzyme catalyst usage in extreme temperature environments. In addition, we assessed the protective effect of HOF-PTBA under harsh conditions, including heavy metal ions and denaturing reagents. As depicted in Fig. 4c, Cyt-c@HOF-PTBA retained at least 65% activity in the presence of Fe(CN)_6_^3+^, Ni^2+^, Cu^2+^, and Cr^3+^. Moreover, Cyt-c@HOF-PTBA exhibited robust resistance to urea, maintaining 92% catalysis activity (Fig. 4d).

The exceptional stability of Cyt-c@HOF-PTBA prompted us to assess its activity without additional protection, which could offer significant advantages in storage and transportation (Fig. S26†). We conducted an evaluation by incubating Cyt-c@HOF-PTBA at 35 °C for 1–8 days and subsequently testing its catalytic activity. Remarkably, experimental results revealed that HOF-PTBA maintained 72% catalytic activity even after 8 days (Fig. S27†). These results encouraged us to explore the reusability of the Cyt-c@HOF-PTBA biocatalyst, aiming to reduce overall costs and facilitate industrial catalysis. The catalytic ability of Cyt-c@HOF-PTBA remained nearly unchanged after five cycles, retaining 71% of its original activity even after 10 cycles (Fig. S28†). The slight decrease in catalytic activity during the recycling assay may be due to the loss of Cyt-c@HOF-PTBA during centrifugation and filtration steps. These results underscore the remarkable attributes of Cyt-c@HOF-PTBA, including high catalytic activity, durability, reusability, and resistance to harsh environments. Thus, HOF-PTBA represents an ideal model for enzyme immobilization, highly desirable for long-term applications in enzyme catalysis.

**Fig. 5:**
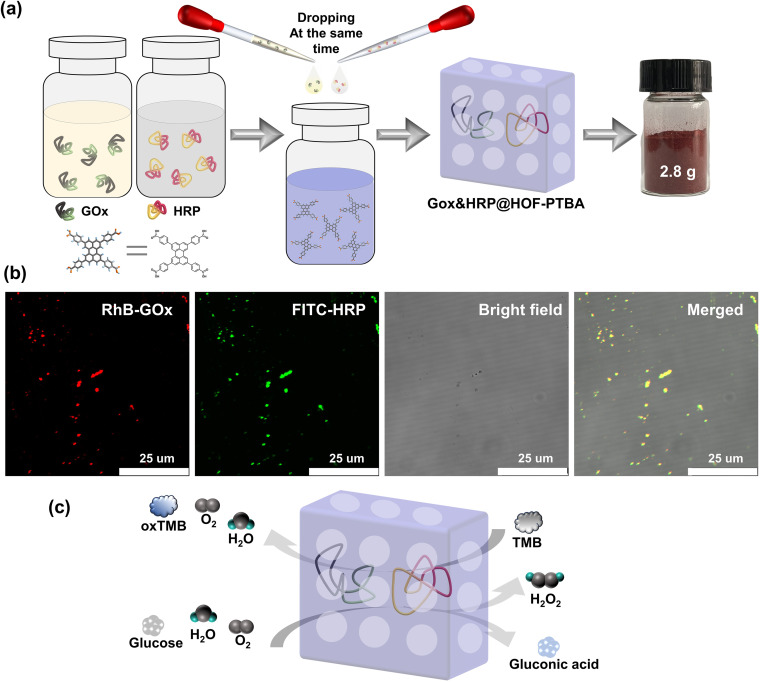
(a) Facile synthesis of GOx&HRP@HOF-PTBA at room temperature and a digital photograph based on a scale-up synthesis (2.8 g for one reaction). (b) CLSM images of GOx&HRP@HOF-PTBA (GOx was labelled with red dye and HRP was labelled with green dye). (c) Cascade reaction mechanism using GOx&HRP@HOF-PTBA as the catalyst.

Encouraged by the results, we proceeded to assess the universality of immobilization supports and extended it to the encapsulation of multiple enzymes. The multienzyme biocatalytic cascade (MBC) plays crucial roles in living organisms, serving functions such as signal transmission and metabolic pathways within cells.^67^ Immobilizing multiple enzymes within the same framework can emulate cell-like spatial isolation,^68^ simulating complex cellular environments with sequential intercommunication and instant signal feedback, thereby enabling specific organism functions. The large pore structure of mesoporous HOF-PTBA facilitates the transport of catalytic substrates, allowing us to achieve a biocatalytic cascade reaction by loading GOx and HRP. *In situ* encapsulation dual-enzyme GOx and HRP within HOF-PTBA can be easily obtained by mixing and stirring them under ambient conditions for 5 minutes, resulting in the production of 2.8 g GOx&HRP@HOF-PTBA biocatalysts through centrifugation (Fig. 5a). As shown in the CLSM images (Fig. 5b), the encapsulated GOx and HRP enzymes were uniformly distributed in the composites, with GOx and HRP labelled with RhB and fluorescein isothiocyanate (FITC), respectively. The PXRD patterns showed similar peaks between GOx&HRP@HOF-PTBA and HOF-PTBA (Fig. S29†), indicating the intact crystalline mesoporous framework during the immobilization reactions. In the FT-IR spectra (Fig. S30†), the presence of similar amide I peaks at 1700–1610 cm^−1^ and amide II peaks at 1590–1485 cm^−1^ in GOx&HRP@HOF-PTBA confirms the successful encapsulation of GOx and HRP within HOF-PTBA. The enzyme loadings of GOx and HRP in the biocomposite were determined to be 11.3% and 11.5% based on the ICP-MS method (Table S5†), respectively. The loading amounts of GOx and HRP in the biocomposite were also quantitatively confirmed by fluorescence changes in the supernatant before and after encapsulation (Fig. S31–S34†), giving values of 11.2% and 11.9%, respectively (Table S6†). After soaking GOx&HRP@HOF-PTBA in buffer solution for 10 days, no enzyme leaching was observed in the solution (Fig. S35†). The TGA curve of GOx&HRP@HOF-PTBA indicated that the pyrolysis of the dual-enzyme occurred at around 250–450 °C (Fig. S36†).

**Fig. 6:**
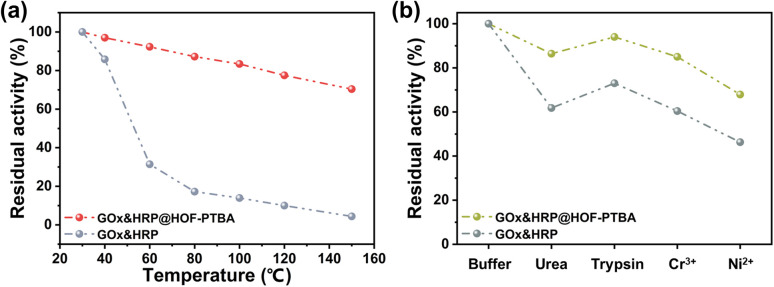
Residual activity of GOx&HRP@HOF-PTBA after treatment by heating for 30 min (a) and using denaturing reagents and heavy metal ions (b).

We further investigated the biocatalytic cascade performance of GOx&HRP@HOF-PTBA. The dual-enzyme cascade reactions involve the oxidation of glucose solution in the presence of GOx, yielding H_2_O_2_ and gluconic acid, with H_2_O_2_ subsequently acting as a substrate for HRP to catalyze the oxidation of TMB to oxidized TMB (oxTMB) (Fig. 5c). The quantity of blue oxTMB products can be evaluated using UV-vis spectroscopy (Fig. S37†). We assessed the feasibility of *in situ* encapsulation of the dual enzymes by comparing the catalytic activity of GOx&HRP@HOF-PTBA with that of the GOx&HRP mixture under different temperature conditions, pH levels, metal ions, and denaturing reagents. Compared to free GOx&HRP, GOx&HRP@HOF-PTBA exhibited notable advantages in residual activity with increasing reaction temperatures (Fig. 6a); the higher the temperature, the bigger the gap. When the reaction temperature is over 80 °C, the residual activity of the GOx&HRP mixture was lower than 20%, meanwhile that of GOx&HRP@HOF-PTBA was still close to 90% (Fig. S38†). Regarding pH effects, GOx&HRP@HOF-PTBA demonstrated higher residual activity under acidic (pH = 2) and neutral (pH = 7) conditions (Fig. S39†). In the presence of deactivating reagents such as urea, trypsin, Cr^3+^ and Ni^2+^, the enzyme activity of GOx&HRP@HOF-PTBA was significantly higher (21–25%) than that of free GOx&HRP, because of the rigid encapsulation support of HOF-PTBA (Fig. 6b and S40†). In addition, the performance of GOx&HRP@HOF-PTBA surpassed that of free GOx&HRP in organic solvent environments (Fig. S41 and S42†). Long-term storage stability at room temperature was evaluated by measuring residual activities daily (Fig. S43†). While the activity of free GOx&HRP decreased considerably and was only 24% after 12 days, GOx&HRP@HOF-PTBA retained approximately 61% of its initial activity after 12 days (Fig. S44†). These results clearly demonstrate that GOx&HRP@HOF-PTBA possesses enhanced stability and higher activity, validating the advantages of the *in situ* encapsulation strategy.

## Conclusion

In summary, we have presented a facile and versatile enzyme encapsulation strategy for scaling up high-performance enzyme@meso-HOF biocatalysts. The ultrastable mesoporous HOF-PTBA was fabricated by selecting building units with larger conjugated π-systems, which could remain intact even under harsh conditions such as 10 M HNO_3_ and pH = 13. Furthermore, HOF-PTBA could effectively protect mono- and dual-enzymes during the initial growth phase, enabling the retention of biocatalytic activity after encapsulation. Enzymes confined within the ordered and robust HOF-PTBA network exhibited enhanced stability and maintained their biological activity even after exposure to harsh treatments, including heating, organic solvents, metal ions, denaturing reagents, and acid–base solutions. Significantly, the scaled-up syntheses of biocatalysts can be conveniently achieved under mild conditions (aqueous solution and room temperature), aligning perfectly with the principles of green and environmentally friendly production. This study not only overcomes the key bottleneck of current enzyme immobilization technology, but also paves the way for the preparation of high-performance biocatalysts and offers a platform for enzyme industrialization.

## Data availability

The data supporting this article have been included as part of the ESI.† Crystallographic data for HOF-PTBA have been deposited at the CCDC under accession number 2351559 and can be obtained from the CCDC website.

## Author contributions

Z. D. conceived the idea, designed the experiments, wrote the manuscript and provided financial support. Y. Q. and X.-X. Y. performed material synthesis and characterization. H.-R. L. helped with the material synthesis and data analysis. M.-X. Z. and T.-T. R. helped with the characterization and data analysis. C.-P. L. and Y. Z. supervised the experiments, wrote the manuscript and provided financial support.

## Conflicts of interest

There are no conflicts to declare.

## Supplementary Material

SC-OLF-D4SC04619F-s001

SC-OLF-D4SC04619F-s002
