# Supplementary material for: Facile and scale-up syntheses of high-performance enzyme@meso-HOF biocatalysts
Source: Chem Sci. 2024 Sep 20;15(41):16987–96. doi: 10.1039/d4sc04619f (PMC11440381; doi:10.1039/d4sc04619f)
Supplement: SC-015-D4SC04619F-s001 [file SC-015-D4SC04619F-s001.pdf]

Supplementary Information (SI) for Chemical Science.
This journal is © The Royal Society of Chemistry 2024

Supplementary Information

Facile and Scale-Up Syntheses of High-Performance Enzyme@meso-HOF Biocatalysts

Zhengyi Di[1], Yu Qi[1], Xin-Xin Yu[1], Hai-Ruo Li[1], Meng-Xuan Zuo[1,2], Tian-Tian Ren[1], Cheng-Peng Li*[1], and Yanli Zhao*[2]

[1] College of Chemistry, Tianjin Key Laboratory of Structure and Performance for Functional Molecules, Tianjin Normal University, Tianjin 300387

[2] School of Chemistry, Chemical Engineering and Biotechnology, Nanyang Technological University, 21 Nanyang Link, Singapore 637371

*Corresponding Author(s): Cheng-Peng Li, hxxylcp@tjnu.edu.cn; Yanli Zhao, zhaoyanli@ntu.edu.sg

Reagent and materials

The enzymes including cytochrome c (Cyt c, from pig caballus heart), horseradish peroxidase (HRP), glucose oxidase (GOx), and other materials including glucose, lactose and TMB were obtained from Meryer (Shanghai) Biochemical Technology Co., Ltd. The fluorescence dye of fluorescein isothiocyanate (FITC) and rhodamine b isothiocyanate (RhBTC) was purchased from Macklin Biochemical Technology Co., Ltd. (Shanghai, China). Unless otherwise mentioned, all reagents and solvents were purchased from commercial sources and used as received without further purification.

Characterization

^{1}H NMR spectra were recorded on a Bruker AV 400 spectrometer, and the chemical shifts were reported in ppm with respect to reference standards (Figure S2-4). The solid-state NMR measurements were carried out on a Bruker Avance Neo 400WB spectrometer. Single crystal X-ray diffraction data was collected at 100 K on an Oxford Diffraction SuperNova diffractometer equipped with Cu-Kα radiation (λ=1.5418 Å). FT-IR spectra were taken on a Bruker AlPHA FT-IR spectrometer (with KBr pellets) in 4000–400 cm^{-1}. EPR experiment was carried out on Bruker EMX plus 6/1. The test temperature was set at 100 K. Powder X-ray diffraction (PXRD) patterns were recorded on a Bruker D8 Advance diffractometer (Cu-Kα, γ = 1.5406 Å), operating at 40 kV and 100 mA, and the diffraction intensity data were obtained by the continuous scans in the 2θ/θ mode with scan speed of 2 s/step and step size of 0.02°. The scanning transmission electron microscope (STEM) and energy-dispersive X-ray spectroscopy (EDS) were performed on a Thermo Fisher Scientific Talos F200X scanning electron microscope, with the electron beam energy of 200 keV. Thermogravimetric analysis (TGA) plots were recorded on a TGA Q500 thermal analyzer in 25–800 °C at a heating rate of 10 °C/min under N_2 atmosphere. The ultraviolet-visible (UV-Vis) absorbance measurements were performed with a 2800S spectrophotometer (SOPTOP, Shanghai). Confocal laser scanning microscope (LSM 880 NLO, Carl Zeiss, Göttingen, Germany) was used to determine the distribution of RhB-labelled Cyt c within the nanosystem. The O_2 produced by Cyt-c@HOF-PTBA in solution was monitored with a Mettler Toledo Dissolved Oxygen Meter S9 -Standard Kit. The Fe^{3+} concentrations were determined on a Perkin-Elmer Avio 200 inductively coupled

plasma optical emission spectrometer (ICP-OES).

Syntheses

Synthesis of 2,5,8,11-tetrakis(4,4,5,5,-tetramethyl-1,3,2-dioxaboranyl)perylene. Perylene (477.2 mg, 1.885 mmol) and bis pinacolato diboron (2.891 g, 11.38 mol) were added to a 250 mL Schlenk flask equipped with a stirrer. The flask was then evacuated and refilled with N_2 three times before transferring to a glovebox. Inside the glovebox, $[Ir(OMe)(COD)]_2$ (137 mg, 0.207 mmol) and 3,4,7,8-tetramethyl-1,10-phenanthroline (63.4 mg, 0.268 mmol) were added. Dry THF (38 mL) was added outside the glovebox via syringe and the resulting mixture was stirred at 70 °C under N_2 atmosphere for 24 h. The flask was wrapped with aluminum foil during the reaction. After completion, the reaction mixture was cooled to room temperature and solvent was removed under reduced pressure. The resulting solid was purified by column chromatography in DCM to give a yellow solid (0.992 g, 70% yield). ^1H NMR (400 MHz, $CDCl_3$, ppm): δ 8.63 (s, 4H) 8.25 (s, 4H) 1.43 (s, 48H).

Synthesis of 2,5,8,11-tetrakis(n-butyl-benzoate-4-yl)perylene. 2,5,8,11-Tetrakis(4,4,5,5,-tetramethyl-1,3,2-dioxaboranyl)perylene (535.7 mg, 0.7084 mmol), $Pd_2(dba)_3$ (67.7 mg, 0.0739 mmol), triphenyl phosphine (77.7 mg, 0.296 mmol), CsF (430.9 mg, 2.837 mmol), Cs_2CO_3 (1.146 g, 3.517 mmol), and ethyl 4-iodobenzoate (7 g, 25.36 mmol) were dissolved in a solvent mixture of THF (40 mL) and DMF(40 mL) in a 250 mL glass bomb. This mixture was wrapped in aluminum foil. The reaction was heated with stirring at 80 °C for 72 h. After completion, the reaction mixture was cooled to room temperature followed by evaporation of organic solvents. The remaining solid was washed by hexanes (1.1064 mg, 57% yield). ^1H NMR (400 MHz, $CDCl_3$, ppm) δ 8.48 (s, 4H) 8.16 (d, J = 8.2 Hz, 8H) 7.98 (s, 4H), 7.83 (d, J = 8.2 Hz, 8H) 4.39 (t, J = 6.6 Hz, 8H) 1.38 (t, J = 6.0 Hz, 12H).

Synthesis of 2,5,8,11-tetrakis(4-carboxyphenyl)perylene (H_4L). 2,5,8,11-Tetrakis(n-butyl-benzoate-4-yl)perylene (317.6 mg, 0.3318 mmol), NaOH (241.5 mg, 6.038 mmol), MeOH (40.0 mL), THF (40.0 mL), and H_2O (40 mL) were added to a 250 mL bomb flask equipped with a stir bar. The resulting mixture was heated with stirring at 80 °C overnight. After completion, the mixture was cooled down to room temperature, and the organic solvents were removed under reduced pressure. The remaining solid was dissolved in H_2O and stirred at room

temperature for an hour followed by filtration. The filtrate was acidified by HCl and the precipitate was collected by centrifuge. Drying under high vacuum overnight gave the product as a red solid (222.9 mg, 92% yield). ^{1}H NMR (400 MHz, DMSO-D$_6$, ppm) δ 13.06 (s, 4H) 8.89 (s, 4H) 8.32 (s, 4H) 8.19 (d, J = 8.2 Hz, 8H) 8.14 (d, J = 8.2 Hz, 8H).

Preparation of HOF-PTBA for single crystal X-ray diffraction analysis: 2,5,8,11-Tetrakis(4-carboxyphenyl)perylene (10 mg, 0.014 mmol) was dissolved in DMF (2 mL) in a 5 mL uncapped vial. The vial was put in a 30 mL beaker containing MeOH (6 mL) and the beaker was sealed with a plastic film. The red block crystals of HOF-PTBA suitable for single crystal X-ray diffraction analysis were grown by slow vapor diffusion for 5 days at room temperature.

Synthesis of powder HOF-PTBA: 2,5,8,11-Tetrakis(4-carboxyphenyl)perylene (100 mg, 0.1365 mmol) was dissolved in DMF (30 mL), to which H_2O (50 mL) was added and stirred for 1 min to afford red block crystals of HOF-PTBA (94.2 mg, Yield: 94.2%).

In situ installation of HOF-PTBA onto Cyt c: The installation method was based on a biomimetic mineralization process. Organic linker, 2,5,8,11-tetrakis(4-carboxyphenyl)perylene (10 mg), was dissolved in DMF (1 mL) by ultrasonic treatment, named as solution A. Cyt c (5 mg, from horse heart) was dissolved in deionized water (9 mL), named as solution B. Subsequently, solution B was poured into solution A rapidly under stirring. The mixed solution was stirred for 5 min. The obtained Cyt-c@HOF-PTBA biocomposite was collected by centrifugation, and washed with deionized water 3 times and ethanol 3 time in sequence.

Synthesize of GOx&HRP@HOF-PTBA biocomposite: Based on the biomimetic mineralization process, the dual enzymes GOx and HRP were encapsulated in situ within HOF-PTBA. Monomeric H_4L (15 mg) was dissolved in DMF (1.5 mL), which was ultrasonicated and named as solution A. HRP (5 mg) was dissolved in deionized water (5 mL), which was named as solution B. GOx (10 mg) was dissolved in deionized water (5 mL), which was named as solution C. The solutions B and C were quickly poured into solution A at the same time, and the mixed solution was stirred for 5 min. The centrifugation was performed to collect the resulting biological composite was GOx&HRP@HOF-PTBA. After washed sequentially with deionised water for three times and ethanol for one time, the composites were dried in a vacuum oven at room temperature or dispersed in deionized water and stored at 4 °C.

Fluorescence labeling

Fluorescence labeling of enzymes was based on the conjugation between the amino of lysine residue of enzymes and the thiocarbamide of RhB and FITC. In brief, enzyme (20 mg) was dispersed into carbonate buffer solution (10 mL, pH=9.0, 0.5 M), followed by adding fluorescence dyes (1 mg). The mixed solution was then stirred in the dark for 12 h. Finally, the dye-labelled enzyme was obtained through ultrafiltration by a centrifugal filter device (molecular weight cut-off MWCO= 8 kDa) 3 times to remove excess reagents and salts.

Measurement of the Cyt c content in the nanosystem

Bradford protein assays. The Cyt-c content in the nanosystem was determined by detecting the difference in enzyme concentration in the supernatant before and after encapsulation by Bradford protein assay. Cyt-c (5 mg) was added to a mixture of DMF (1 mL) and deionized water (9 mL). The Cyt-c sample (800 μL) was taken, followed by deionized water (7.2 mL), and then Coomassie Brilliant Blue G-250 reagent (500 μL) was added. After incubation for 5 min, the solution was collected and detected by UV-Vis spectrophotometer. The concentration of Cyt-c at 595 nm was proportional to Abs.

Inductively coupled plasma-mass spectrometry. Cyt c is a heme protein (ca. 13 KD) that contains one Fe atom per protein. Inductively coupled plasma-mass spectrometry (ICP-MS) was performed to further evaluate the enzyme loading of Cyt-c@HOF-PTBA by examining the content of Fe in the biocomposites. Briefly, Cyt-c@HOF-PTBA (1.5 mg) was digested by concentrated nitric acid (1 mL) at 120 °C for 4 h. The digested supernatant was collected, and the concentration of Fe was quantified by the standard calibration curve. The Cyt-c loading of Cyt-c@HOF-PTBA could be calculated based on the Fe concentration.

Examination of peroxidase activity of Cyt-c@HOF-PTBA

The peroxidase activity of Cyt-c was evaluated using TMB as the substrates:

$$\mathbf{H_2O_2 + TMB \xrightarrow{\textit{Cyt}\text{-c}} H_2O + oxTMB}$$

The concentrations of Cyt-c in each trial including free Cyt-c and Cyt-c@HOF-PTBA

groups were set at 6.67 μg/mL. In a typical test, the free Cyt-c or Cyt-c@HOF-PTBA were dispersed into Tris buffer (1.8 mL, pH 7.5, 50 mM), followed by adding prepared TMB solution (0.2 mL, 3 mg/mL) as the hydrogen donor. Immediately, H_2O_2 (2 mL, 10 mM) was added to activate the catalytic reaction. The generated oxTMB could be traced at 652 nm by a UV-Vis spectrophotometer using a time-scanning mode.

Examination of O_2 production from decomposition of H_2O_2

Cyt-c@HOF-PTBA (1.5 mg) was added to a certain amount of Tris-HCl buffer (50 mM) and bubbled with high-purity nitrogen for 20 min, then H_2O_2 solution (1 M) was added rapidly, and the oxygen production was monitored by a dissolved oxygen meter.

Evaluation of catalytic activity

The Michaelis-Menten (MM) mechanism is widely used to understand the catalytic activities of various enzymes, which describes the relationship between the steady-state enzymatic velocity and the substrate concentration. That is, a substrate binds reversibly with an enzyme to form a complex, which then undergoes unimolecular decomposition to form a product and the enzyme is regenerated for the next cycle at the same time. The rate of product formation [S] on substrate concentration can be characterized by the MM equation:

$$V = \frac{V_{max}[s]}{[s] + K_m}$$

where V_{max} ($[M \cdot s^{-1}]$) is the maximum formation velocity when the catalytic sites of the enzyme are fully occupied by substrate. [s] ([M]) is the initial substrate concentration, and K_m ([M]) is the Michaelis-Menten constant and can be defined as the concentration of substrate at which the reaction rate is half of v_{max}. The Michaelis constant K_M is not affected by the concentration or purity of an enzyme. Its value depends both on the identity of the enzyme and that of the substrate, as well as conditions such as temperature and pH.

Cyt-c@HOF-PTBA (1.5 mg) was added to Tris-HCl buffer (1.8 mL, pH = 7.5, 50 mM), and then TMB solution (0.2 mL, 3 mg mL^{-1}, DMSO) and H_2O_2 solution (2 mL, 1, 5, 10, 50, 80, and 100 mM) were added rapidly. The yield of oxTMB was monitored by UV-vis at 652 nm wavelength.

GOx and HRP loading measurements

The multi-enzyme loadings in HOF-PTBA were evaluated by the fluorescence labelling experiment. To quantify the multi-enzymes in the cascade system, the enzymes were labelled by dyes with different emission wavelengths. In the assembly process, the raw enzymes were replaced by the dye-labelled enzymes, and other steps were as similar as description above. The enzyme loadings were measured by examining the corresponding fluorescence decrease in the supernatant before and after encapsulation via fluorescence spectra. The FITC- and RhB-labeled enzymes was quantified at λ_{em} = 520 nm (488 nm excitation) and 583 nm (540 nm excitation).

Activity of confined biocatalysis cascade

The cascade activities of enzyme systems were evaluated by tracing the oxTMB at 652 nm using UV-vis spectrophotometer. Typically, the biocatalytic cascade assay was conducted in sodium acetate buffer (500 μL, 100 mM, pH = 5) containing GOx&HRP@HOF-PTBA cascade nanoparticles (5 mg), TMB (200 μM) and substrate (6 mM). The catalytic reaction was initiated by adding the glucose. The activity of the biocatalytic cascade reaction was monitored by following the UV spectra of the generated oxTMB (652 nm wavelength).

$$\mathbf{H_2O_2 + TMB \xrightarrow{\text{GOx\&HRP@HOF-PTBA}} H_2O + oxTMB + O_2}$$

pH stability. Buffer solutions (4.8 mL) of different pH (pH = 2, 3, 4, 5, 6, 7) containing glucose (18.016 mg) were placed in 10 mL glass vials, then TMB solution (200 μL, 6 mM) was added separately, and biocomposite GOx&HRP@HOF-PTBA (5 mg) was added to the glass vials containing buffers of different pH respectively to initiate the reaction. The supernatant of the reaction was taken after 10 min to detect its absorbance at 652 nm by UV spectrophotometer.

Thermal stability. Sodium acetate buffer solution (4.8 mL, pH=5, 0.1 M) containing glucose (18.016 mg) was put into a 10 mL glass vial, and then TMB solution (200 μL, 6 mM) and biocomposite GOx&HRP@HOF-PTBA (5 mg) were added, which was put into an oven at different temperatures (30, 40, 60, 80, 100, 120, and 150 °C) for 30 min.

Stability in organic solvents. Different organic solvents (0.5 mL, in methanol, acetonitrile,

and DMF) and sodium acetate buffer solution (4.3 mL, pH=5, 0.1 M) were added to a 10 mL glass vial, and glucose (18.016 g) was weighed and dissolved in the above 10 mL glass vial. Then, TMB solution (200 μL, 6 mM) was added, and GOx&HRP@HOF-PTBA (5 mg) was also added to the above 10 mL glass vial to initiate the reaction. After 1 min, the supernatant of the reaction was taken and the absorbance at 652 nm was detected quickly by UV spectrophotometer.

Storage stability. Biocomposite GOx&HRP@HOF-PTBA (5 mg) was sealed in a sample tube and then left at room temperature for different days (1, 2, 3, 4, 5, 6, 7, 8, 9, 10, 11, and 12 d). Sodium acetate buffer solution (4.8 mL, pH=5, 0.1 M) containing glucose (18.016 mg) was placed into 10 mL glass vials, and then TMB solution (200 μL, 6 mM) and biocomposites GOx&HRP@HOF-PTBA (5 mg) were added separately, which was stored for different numbers of days.

Reusability. Sodium acetate buffer solution (4.8 mL) containing glucose (18.016 mg) was put into a 10 mL glass vial, TMB solution (200 μL, 6 mM) was added, and biocomposite material GOx&HRP@HOF-PTBA (5 mg) was added to the above mentioned 10 mL glass vial to initiate the reaction. The supernatant of the reaction was taken after 10 min and was immediately detected by a UV spectrophotometer at 652 nm. At the end of the reaction, GOx&HRP@HOF-PTBA was recovered, washed twice with deionized water and once with ethanol, and dried under vacuum at room temperature, and so on for 13 times. The catalytic activity of GOx&HRP@HOF-PTBA was tested after cycling different times.

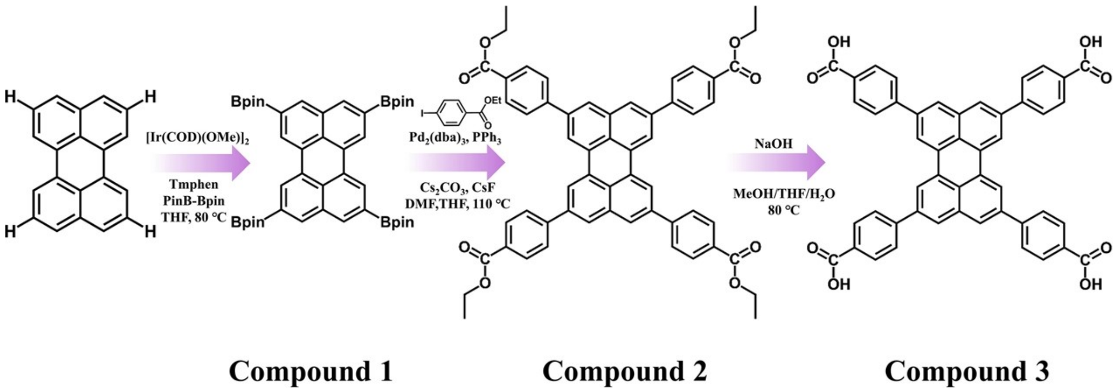

Figure S1. Synthetic routine of the 2,5,8,11-tetrakis(4-carboxyphenyl)perylene (H_4PTBA) ligand.

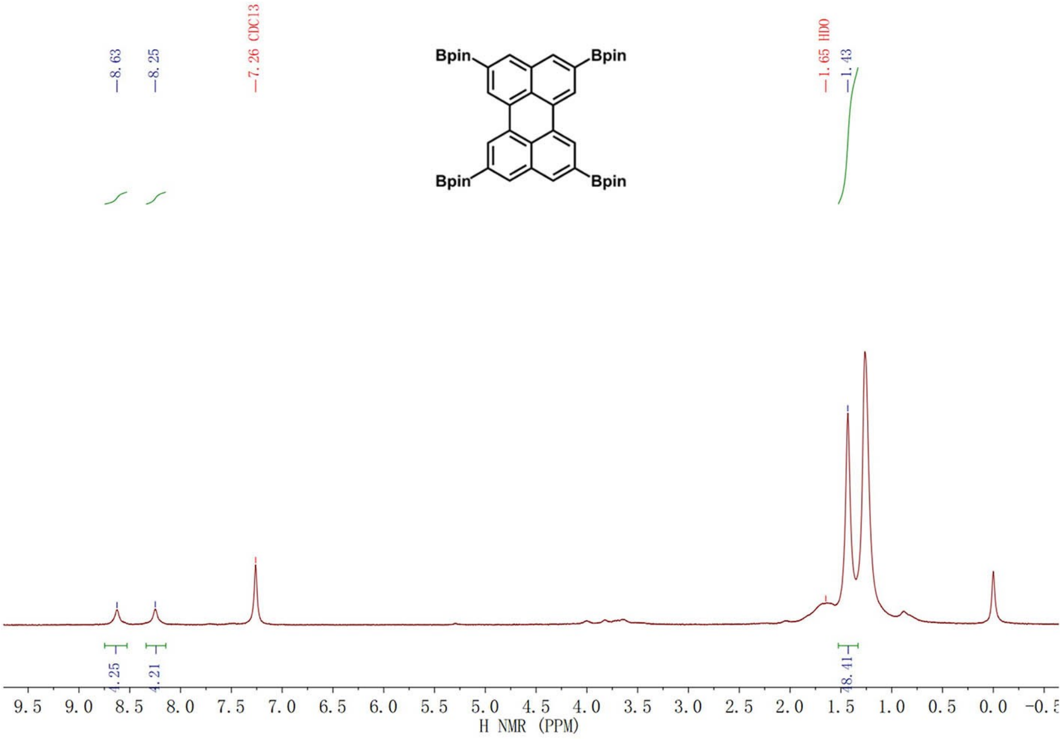

Figure S2. ^{1}H NMR spectrum of 2,5,8,11-tetrakis(4,4,5,5,-tetramethyl-1,3,2-dioxaboranyl)perylene.

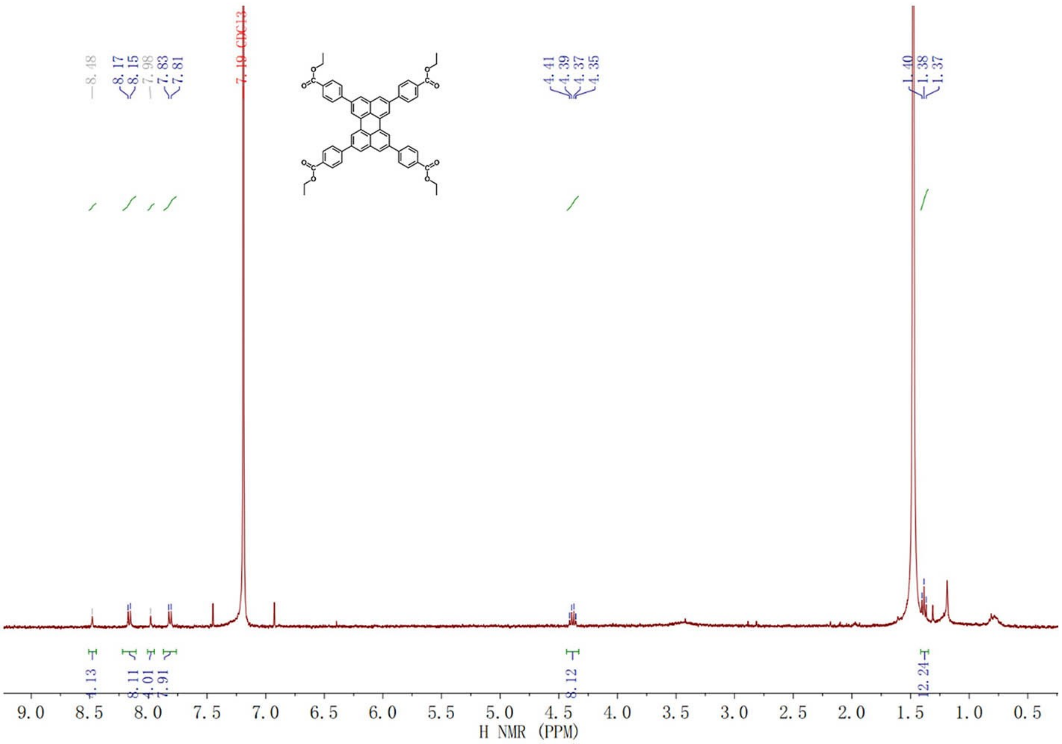

Figure S3. ^{1}H NMR spectrum of 2,5,8,11-tetrakis(n-butyl-benzoate-4-yl)perylene.

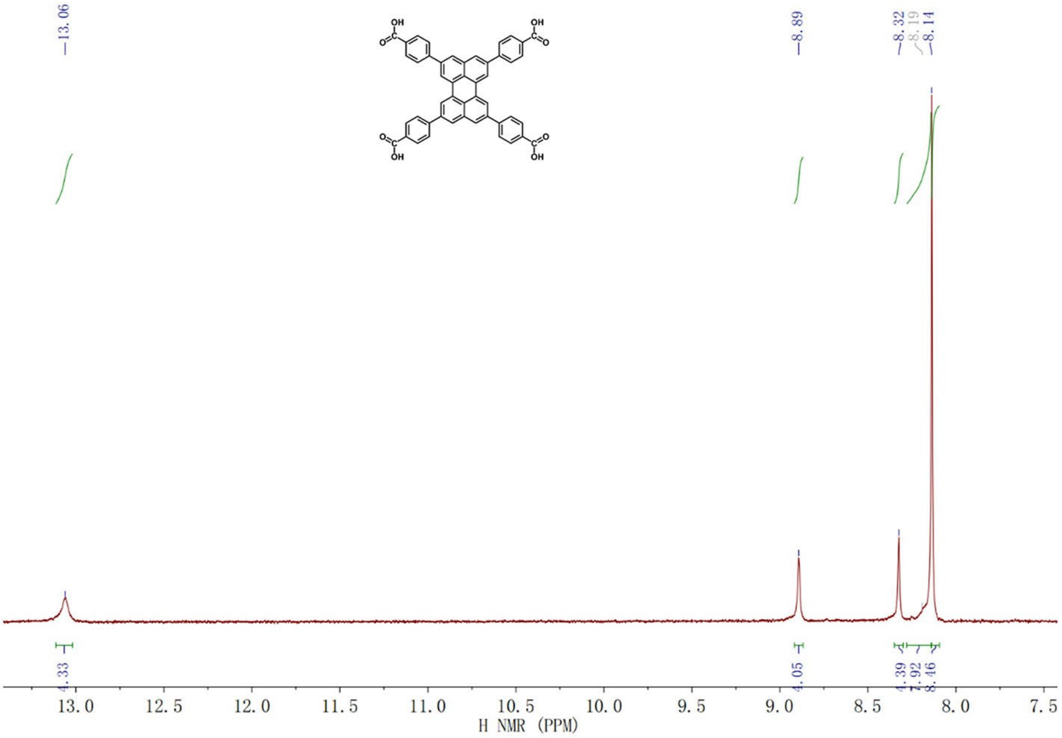

Figure S4. ^{1}H NMR spectrum of 2,5,8,11-tetrakis(4-carboxyphenyl)perylene (H_4PTBA).

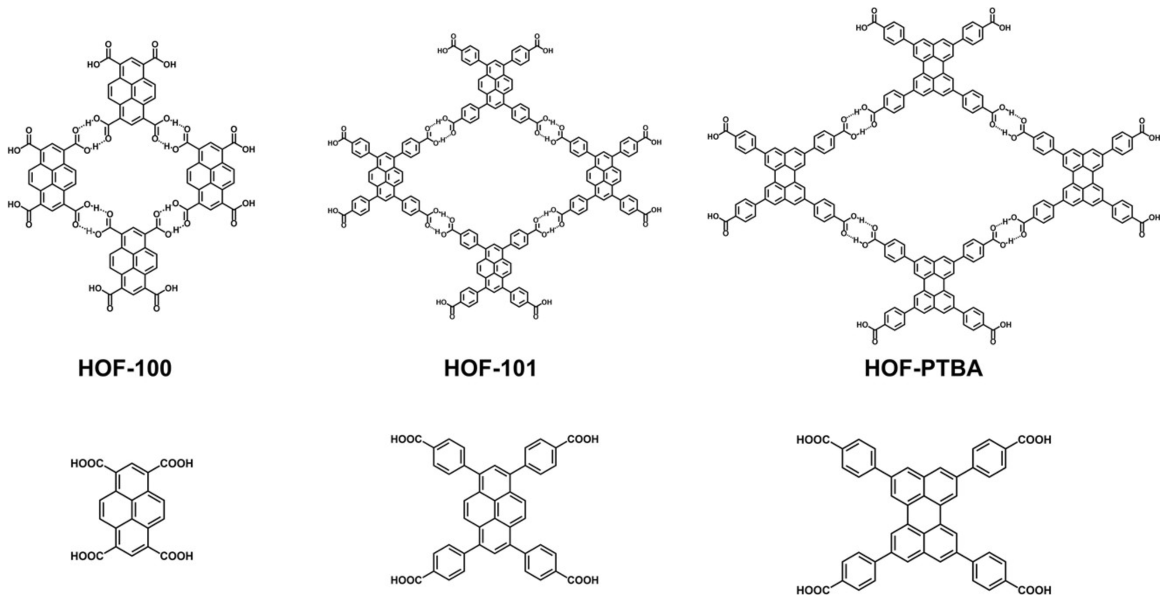

Figure S5. Molecular structures of HOF-100, HOF-101 and HOF-PTBA.

Table S1. Crystal data of HOF-PTBA.

Formula	$C_{12}H_6O_2$
CCDC	2351559
Temperature (K)	100.15
Crystal system	monoclinic
space group	C2/m
a (Å)	22.8640(10)
b (Å)	35.6826(12)
c (Å)	7.4294(2)
α (°)	90
β (°)	91.622(3)
γ (°)	90
V (Å^3)	6058.8(4)
Z	8
F (000)	1504
Completeness	99.3 %
R_1, wR($I > 2\sigma(I)$)[a]	R_1 = 0.0747
	wR_2 = 0.2355
R_1, wR(all data)[b]	R_1 = 0.0976
	wR_2 = 0.2592
GOOF on F^2	1.041

[a]$R_1 = \sum||F_o| - |F_c||/\sum|F_o|$. [b]$wR_2 = \{\sum[w(F_o^2 - F_c^2)^2]/\sum[w(F_o^2)^2]\}^{1/2}$

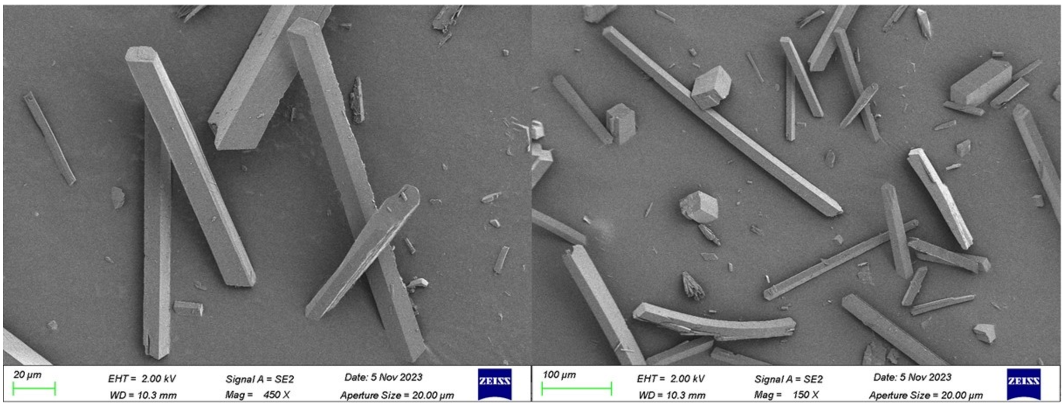

Figure S6. SEM images of HOF-PTBA crystals.

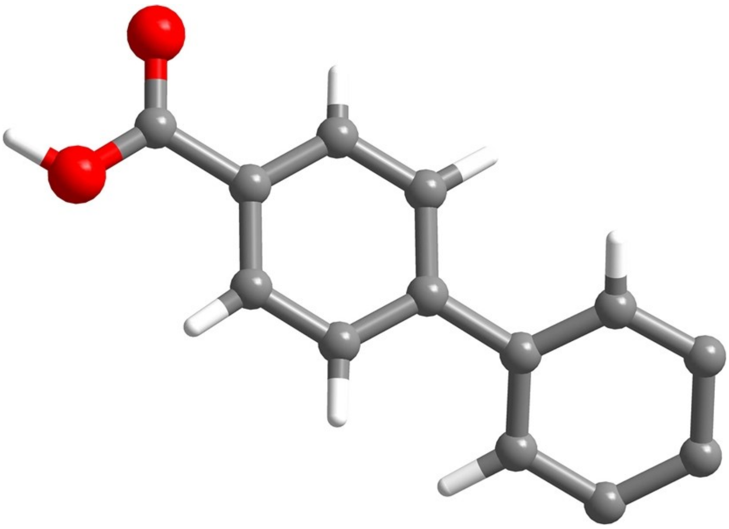

Figure S7. Asymmetric unit of HOF-PTBA.

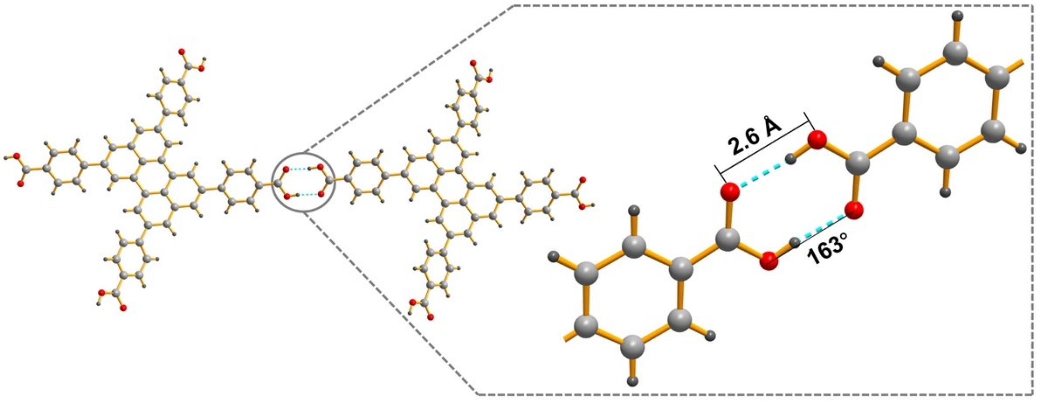

Figure S8. O–H···O bonds in HOF-PTBA, highlighting the bond length and angle.

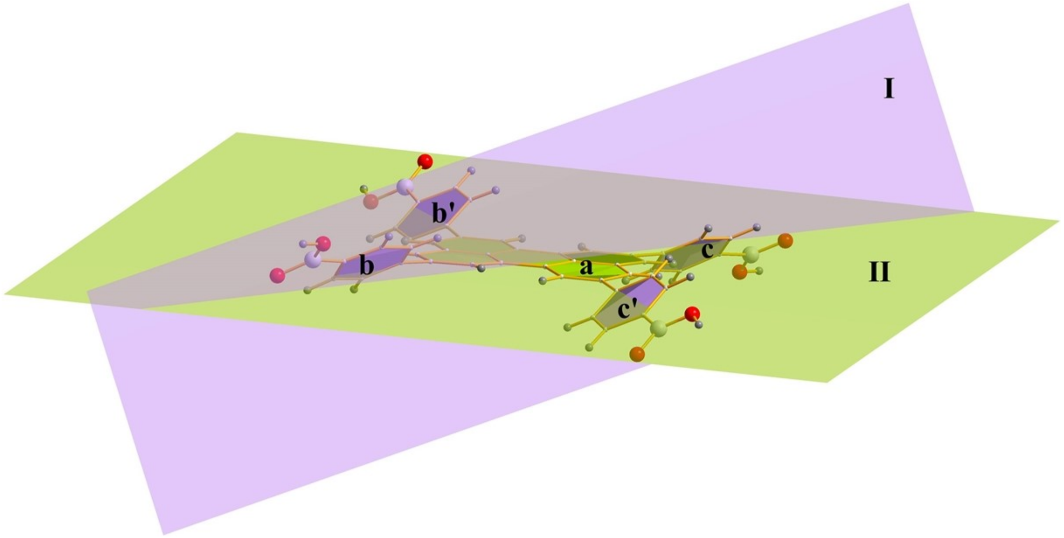

Figure S9. Dihedral angles between the central perylene and side benzene rings in HOF-PTBA.

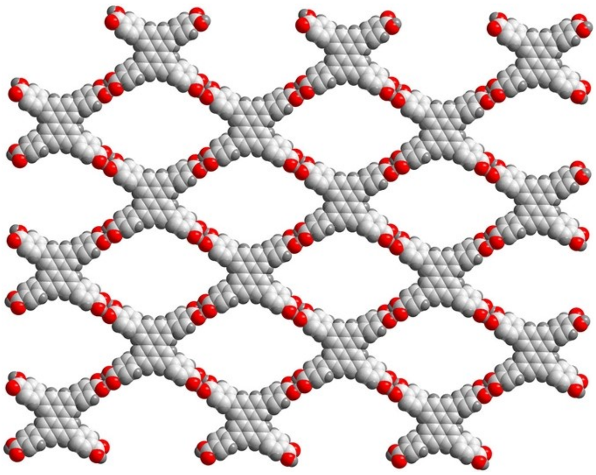

Figure S10. Representation of the porous framework of HOF-PTBA.

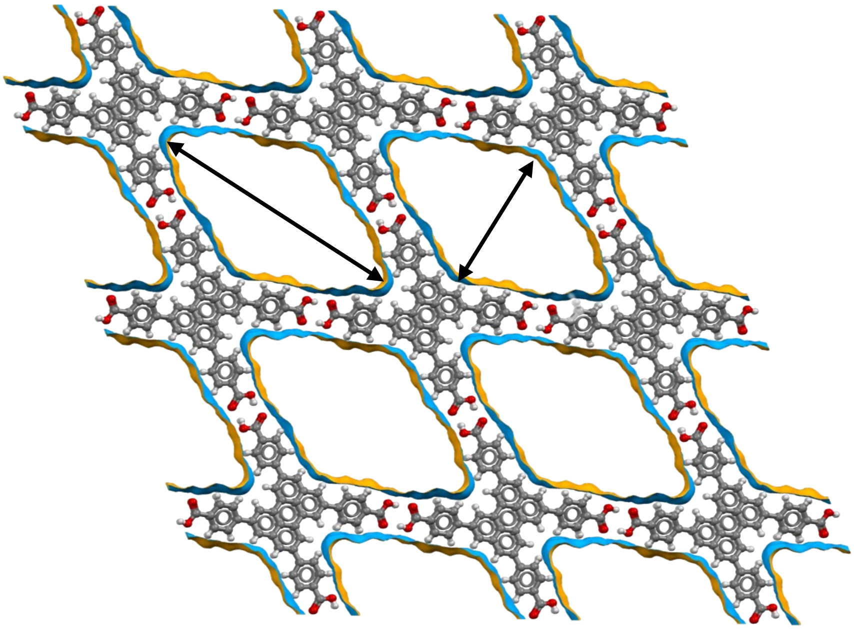

Figure S11. One-dimensional (1D) channels of HOF-PTBA.

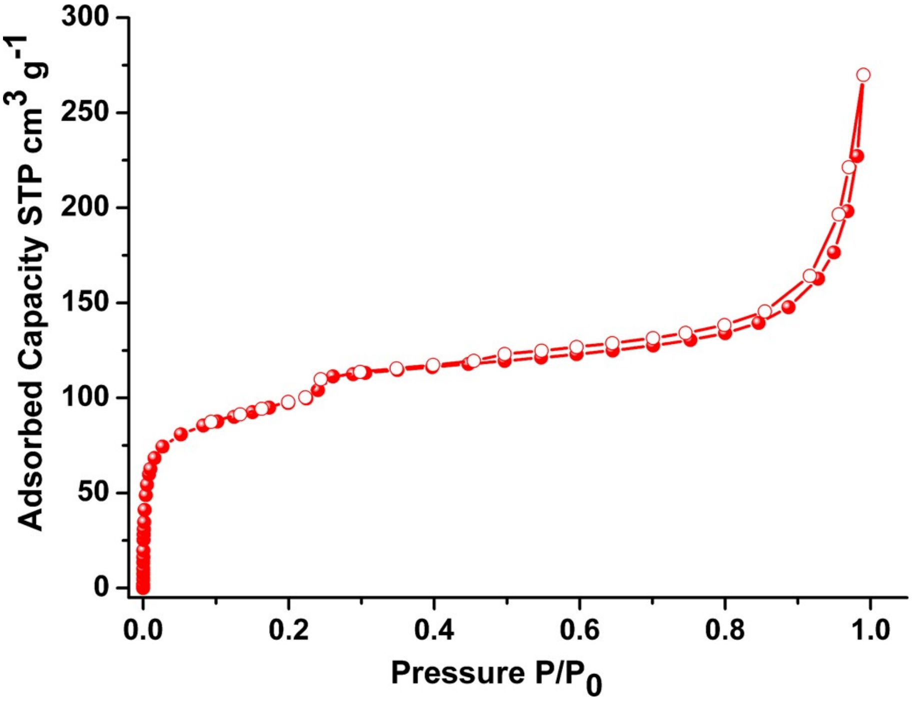

Figure S12. N_2 sorption isotherms at 77 K of HOF-PTBA.

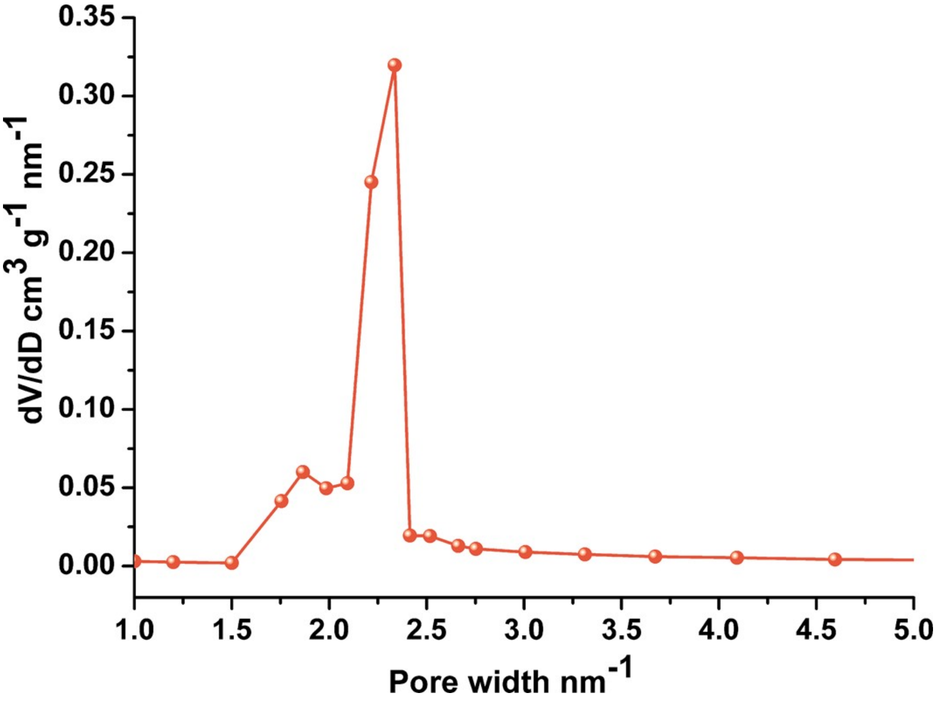

Figure S13. Pore width distribution of HOF-PTBA.

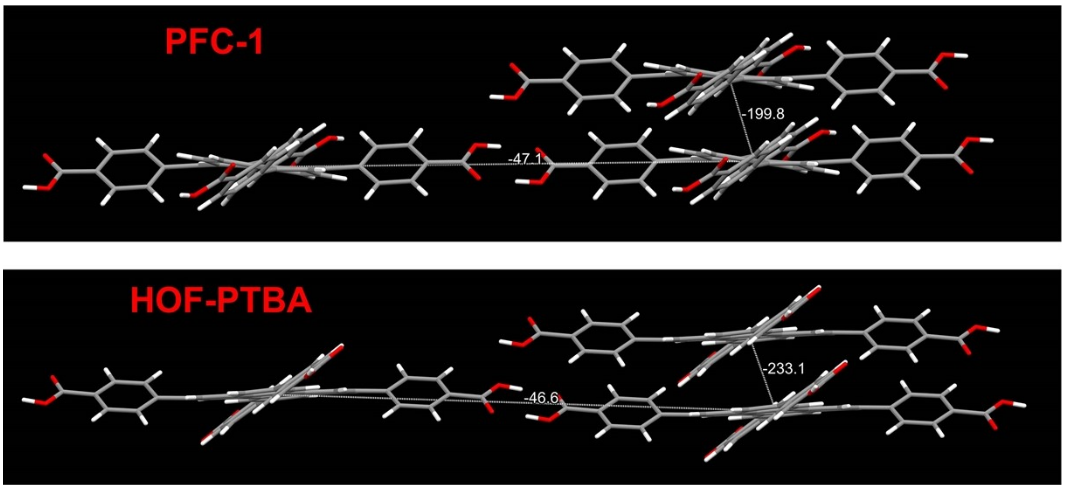

Figure S14. π–π stacking interaction energy and hydrogen bonding energy of PFC-1 and HOF-PTBA dimer. The unit of the value in the diagram is KJ mol^{-1}.

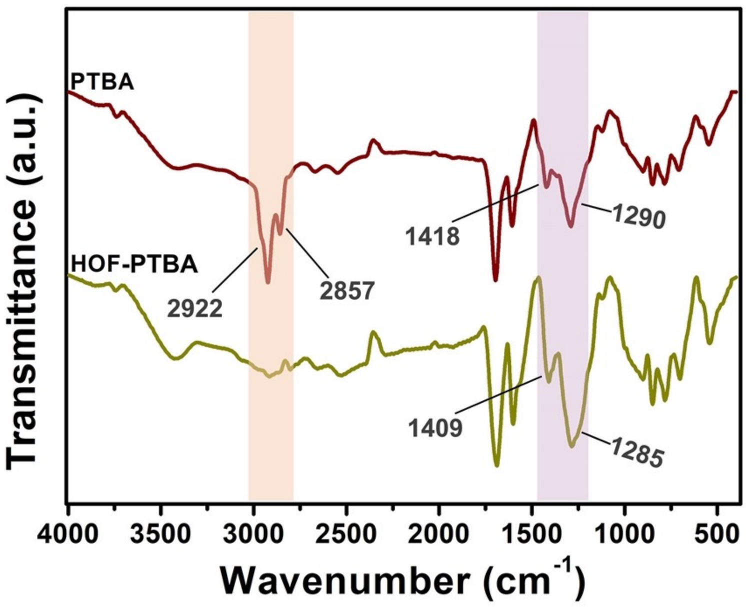

Figure S15. FT-IR spectra of HOF-PTBA and H_4PTBA.

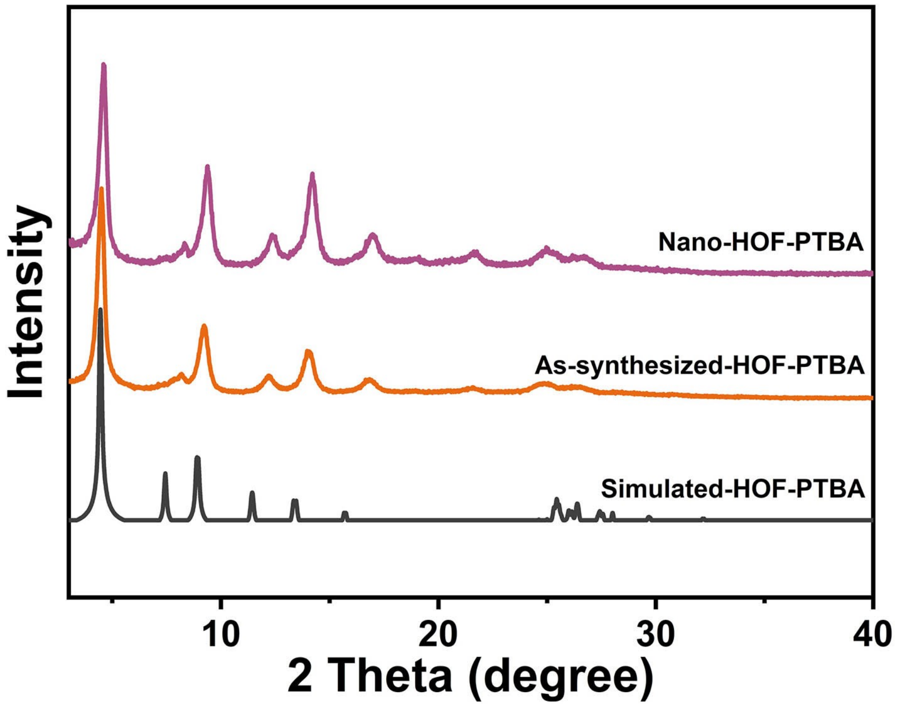

Figure S16. PXRD patterns of simulated HOF-PTBA, as-synthesized HOF-PTBA and powdery HOF-PTBA.

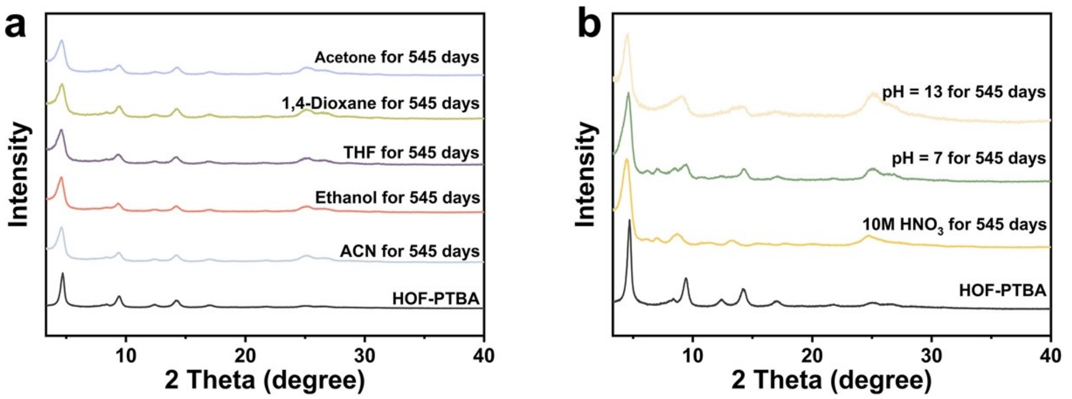

Figure S17. PXRD patterns of HOF-PTBA in different solutions (a) and various pH (b) for 545 days.

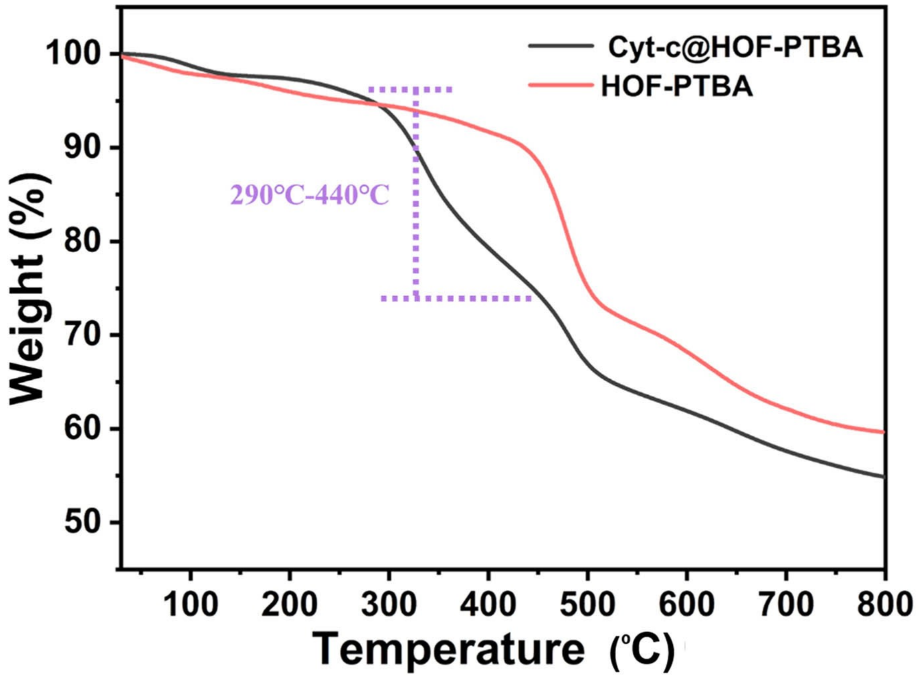

Figure S18. TGA curves of HOF-PTBA and Cyt-c@HOF-PTBA, respectively. The weight loss of Cyt-c@HOF-PTBA between ca. 290 °C to 440 °C was caused by the pyrolysis of the incorporated Cyt c.

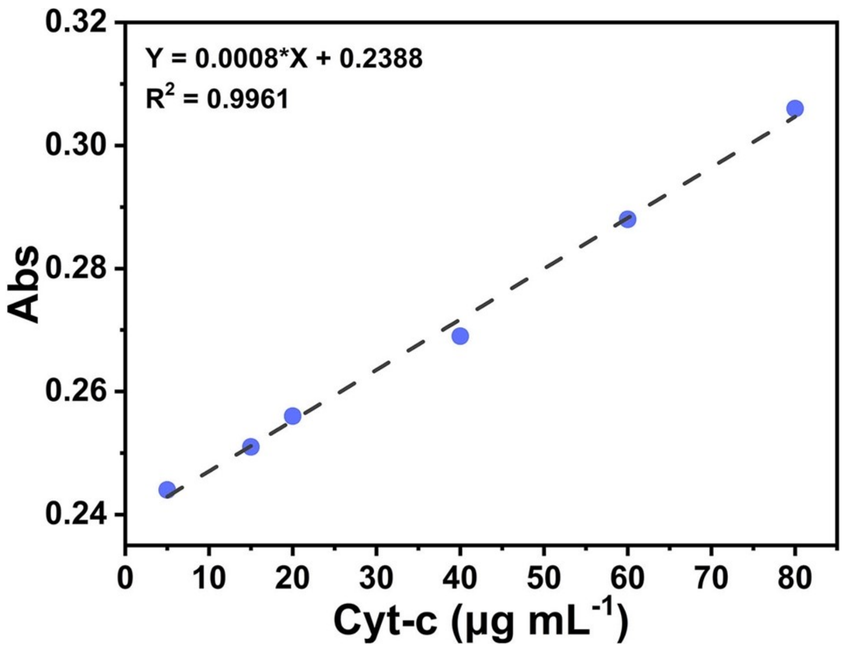

Figure S19. Standard curve for Cyt-c quantification using Bradford proteins assays.

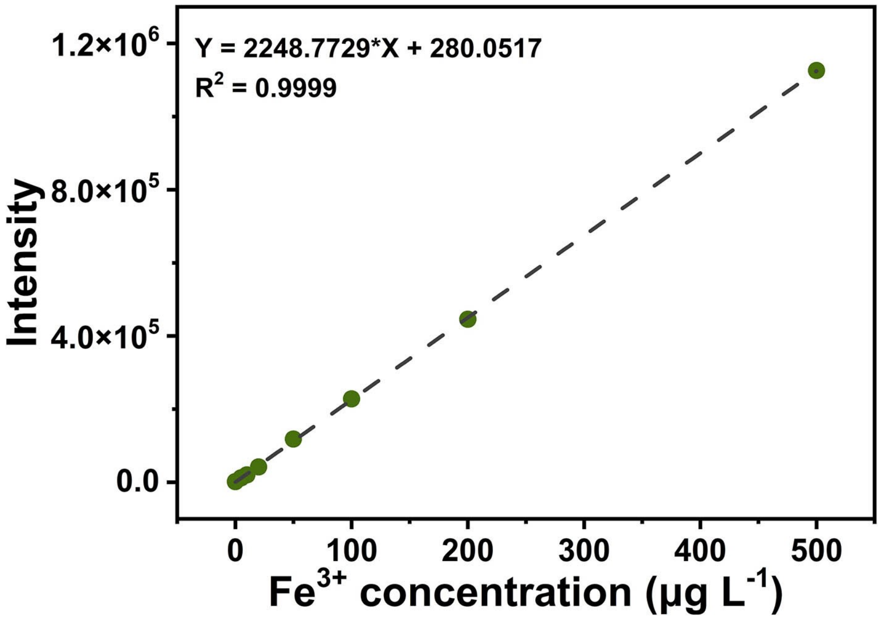

Figure S20. Standard curve of Fe^{3+} ion for Cyt-c quantification using ICP-MS.

Table S2. Calculated Cyt c loadings in three assays of Cyt-c@HOF-PTBA via ICP-MS.

Cyt-c@HOF-PTBA	No. 1	No. 2	No. 3	Average
Fe (wt%)	0.15827	0.1666	0.1642	0.16302
Cyt-c (wt%)	36.8065	38.7442	38.186	37.9122

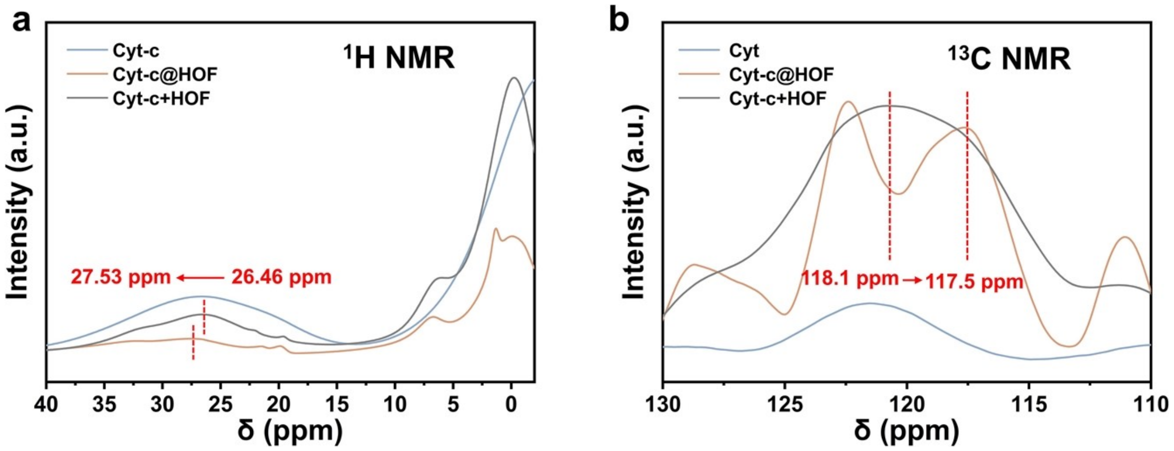

Figure S21. Solid-state ^{1}H NMR (a) and ^{13}C NMR (b) spectra.

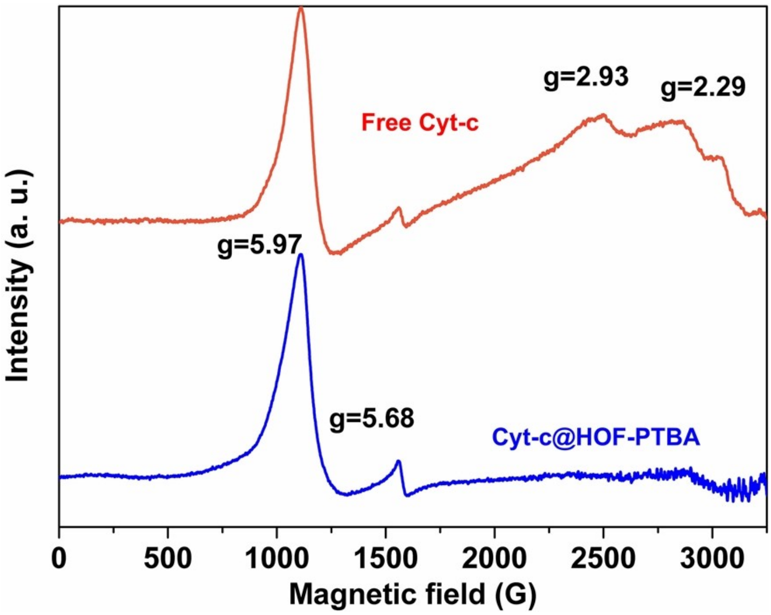

Figure S22. EPR spectra of free Cyt-c and Cyt-c@HOF-PTBA collected at 100 K.

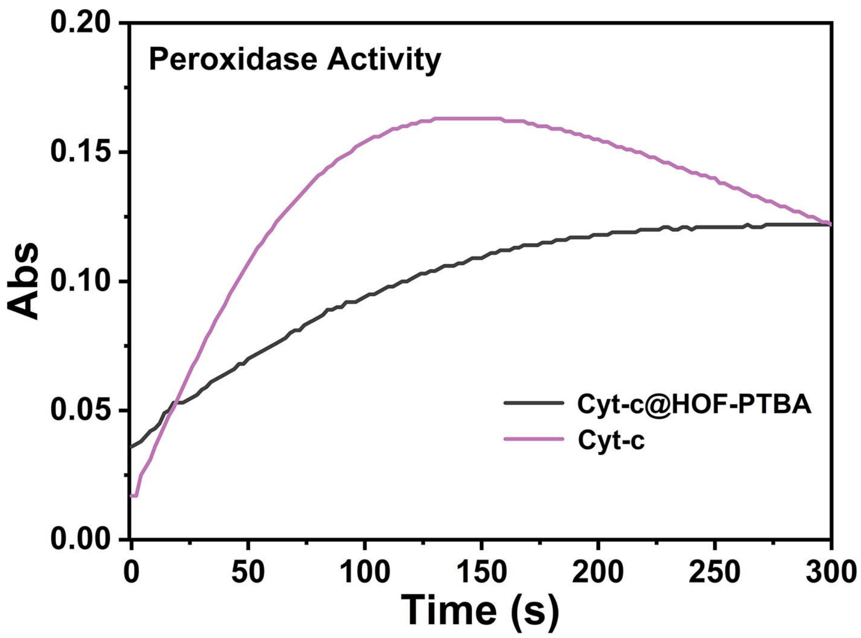

Figure S23. Peroxidase activity of Cyt-c@HOF-PTBA.

Table S3. Comparison of catalysis activity between Cyt-c@HOF-101 and Cyt-c@HOF-PTBA.

	**Cyt-c@HOF-101 O_2 Production (mg/L, 50s)**	**Cyt-c@HOF-PTBA O_2 Production (mg/L, 50s)**	**O_2 (HOF-PTBA) : O_2 (HOF-101)**
**0.1 M H_2O_2**	11.32	22.11	1.95
**0.08 M H_2O_2**	8.51	17.17	2.02
**0.05 M H_2O_2**	5.74	10.65	1.86
**0.001 M H_2O_2**	0.59	1.07	1.81
**pH=2**	2.29	3.69	1.61
**pH=4**	3.28	3.85	1.17
**pH=6**	3.89	4.31	1.11
**pH=8**	3.84	4.11	1.07
**60 ℃**	2.58	3.81	1.48
**80 ℃**	2.57	3.7	1.44
**100 ℃**	2.24	3.5	1.56

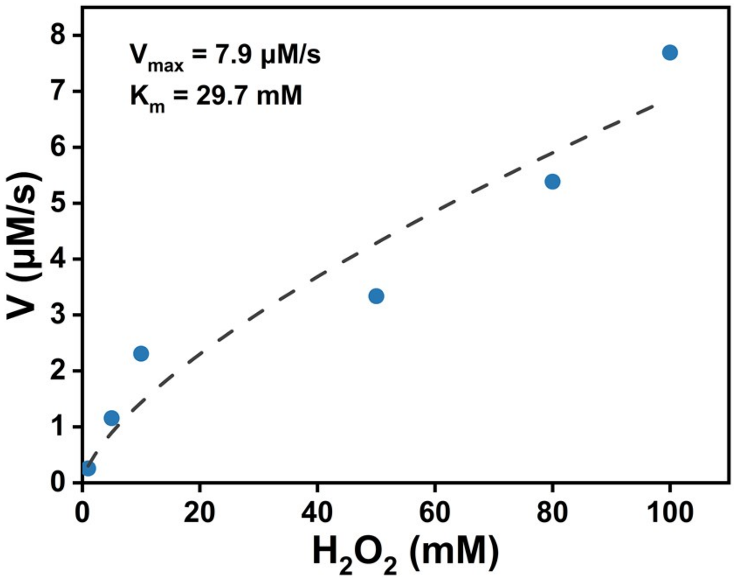

Figure S24. Michaelis-Menten enzymatic kinetics of Cyt-c@HOF-PTBA.

Table S4. Kinetics parameters of Cyt-c@HOF-PTBA and Cyt-c@HOF-101.

	V_{max} (mM/s)	K_m (mM)
**Cyt-c@HOF-PTBA**	**7.9**	**29.7**
**Cyt-c@HOF-101**	**7.2**	**32.3**

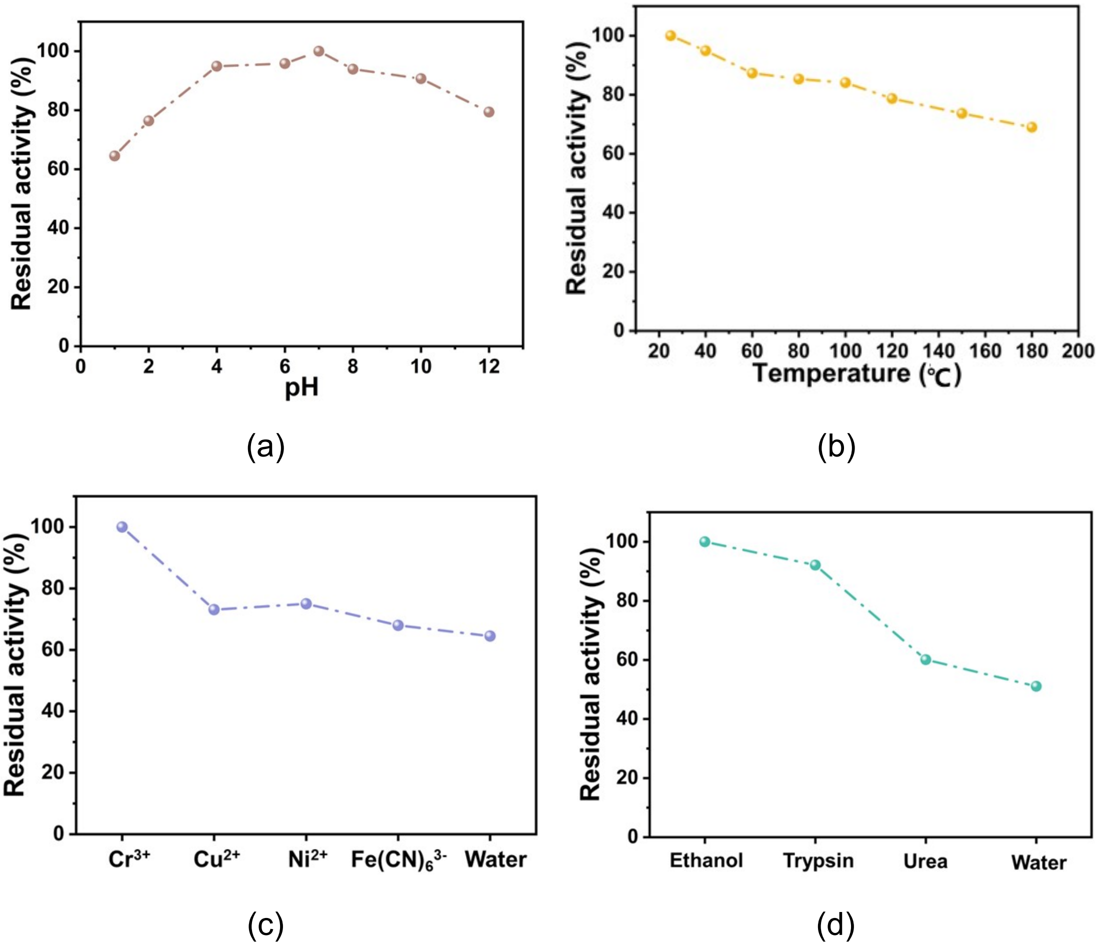

Figure S25. Residual activity of Cyt-c@HOF-PTBA after treatment by non-physiological pH (a), heating for 30 min (b), exposure to heavy metal ions (c), and denaturing reagents and organic solvents (d).

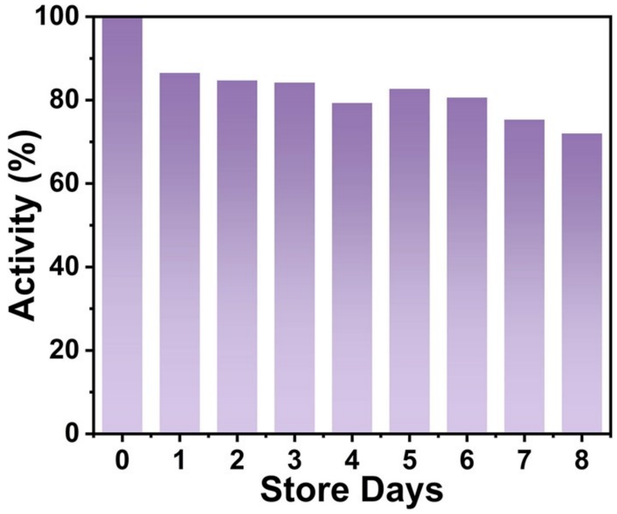

(a)

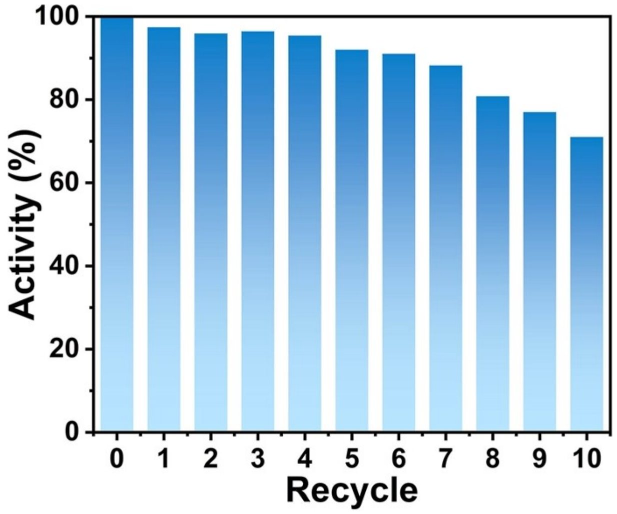

(b)

Figure S26. Residual activity of Cyt-c@HOF-PTBA after treatment by keeping at 35 °C after several days (a) and various recycles (b).

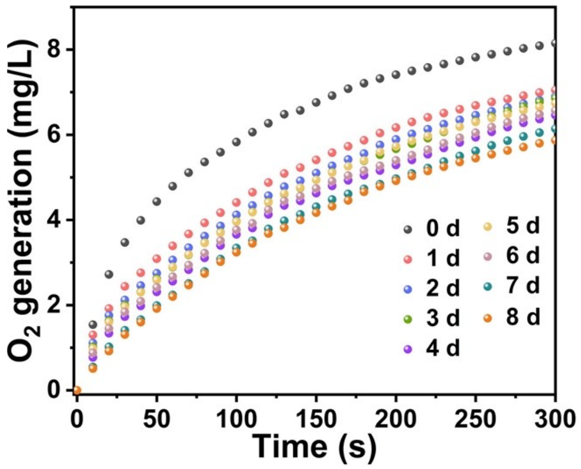

Figure S27. Catalytic kinetics of Cyt-c@HOF-PTBA after keeping at 35 °C for several days.

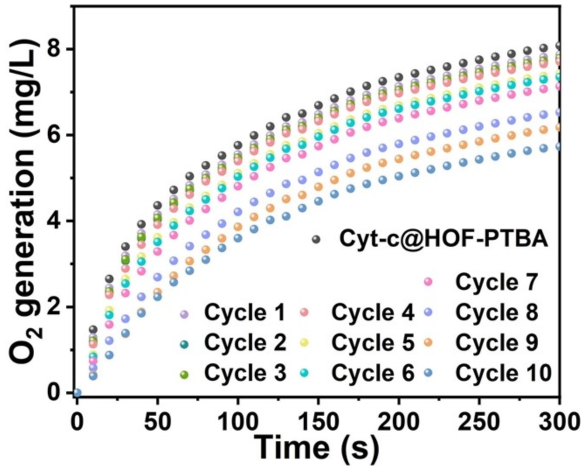

Figure S28. Catalytic kinetics of Cyt-c@HOF-PTBA after various recycles.

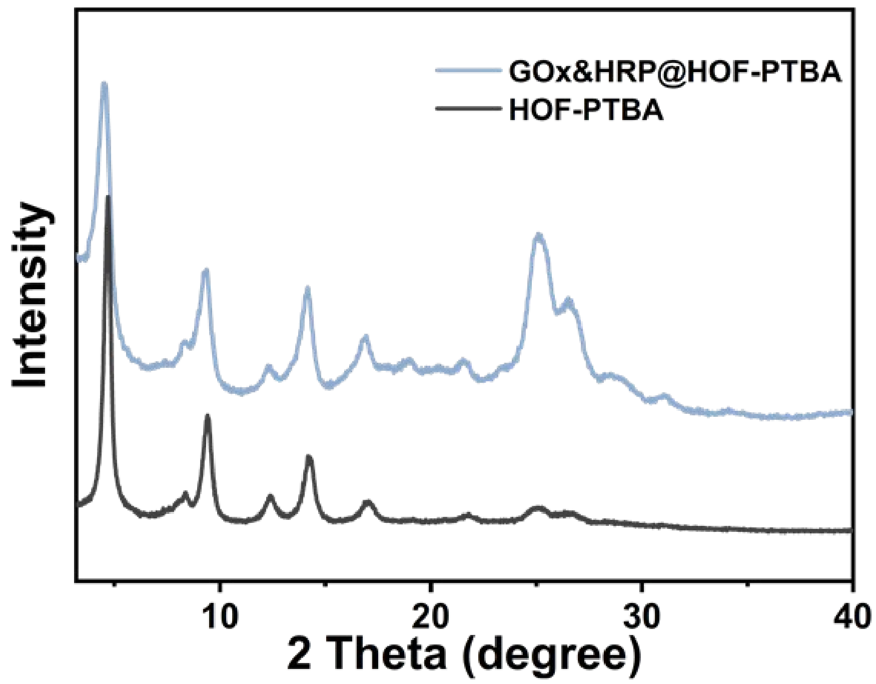

Figure S29. PXRD patterns of GOx&HRP@HOF-PTBA and experimental HOF-PTBA.

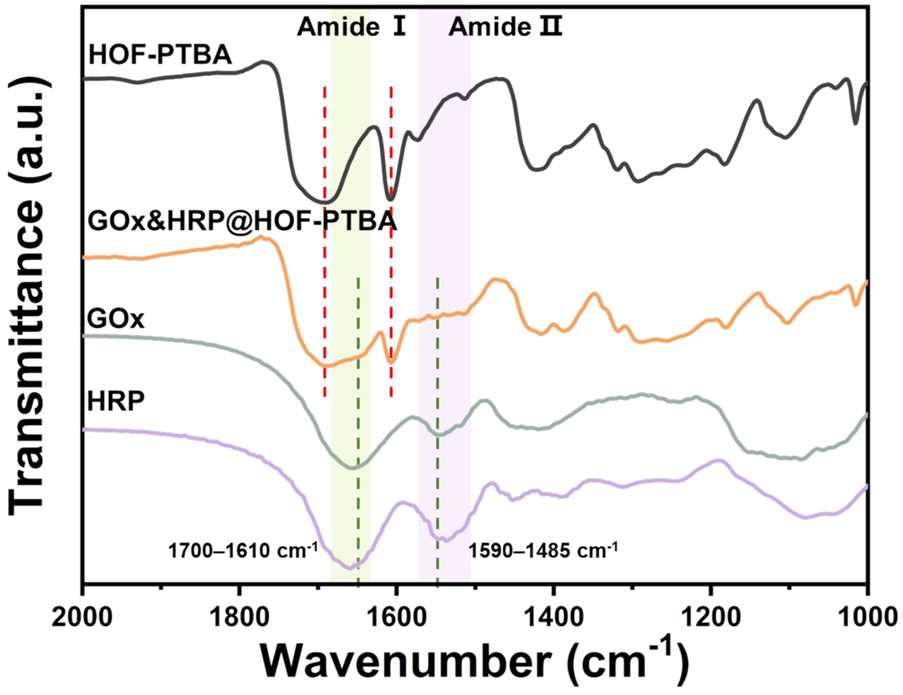

Figure S30. FT-IR spectra of GOx, HRP, GOx&HRP@HOF-PTBA and HOF-PTBA, respectively.

Table S5. Calculated GOx and HRP loadings in three assays of GOx&HRP @HOF-PTBA via ICP-MS.

GOx&HRP @HOF-PTBA	No. 1	No. 2	No. 3	Average
**Fe (wt%)**	0.0351	0.0363	0.0357	0.0357
**HRP (wt%)**	11.331	11.708	11.521	11.520
**GOx (wt%)**	11.489	11.112	11.300	11.301

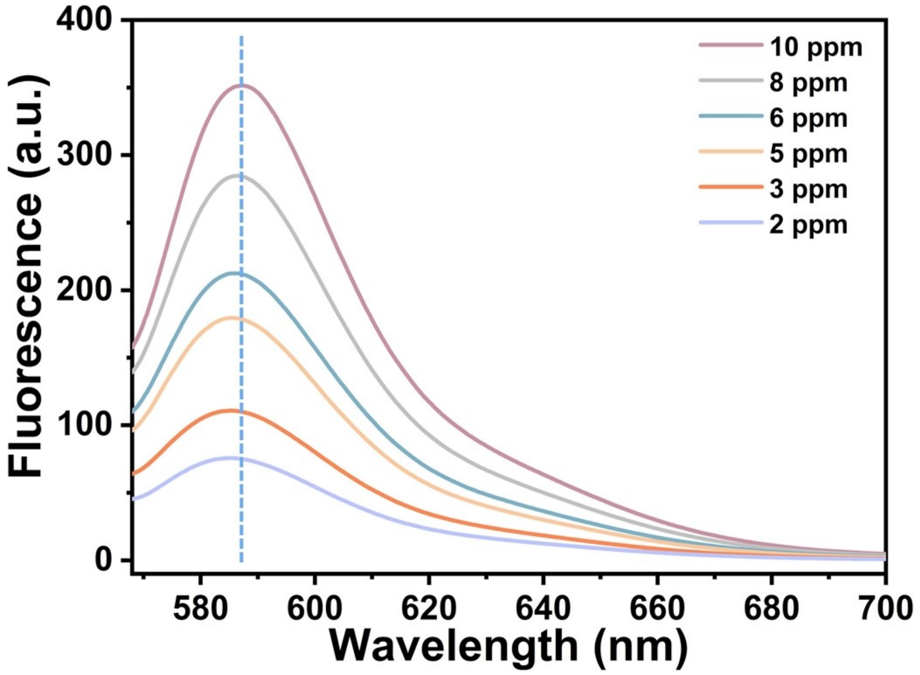

Figure S31. Fluorescence curves of quantitative GOx by fluorescence method.

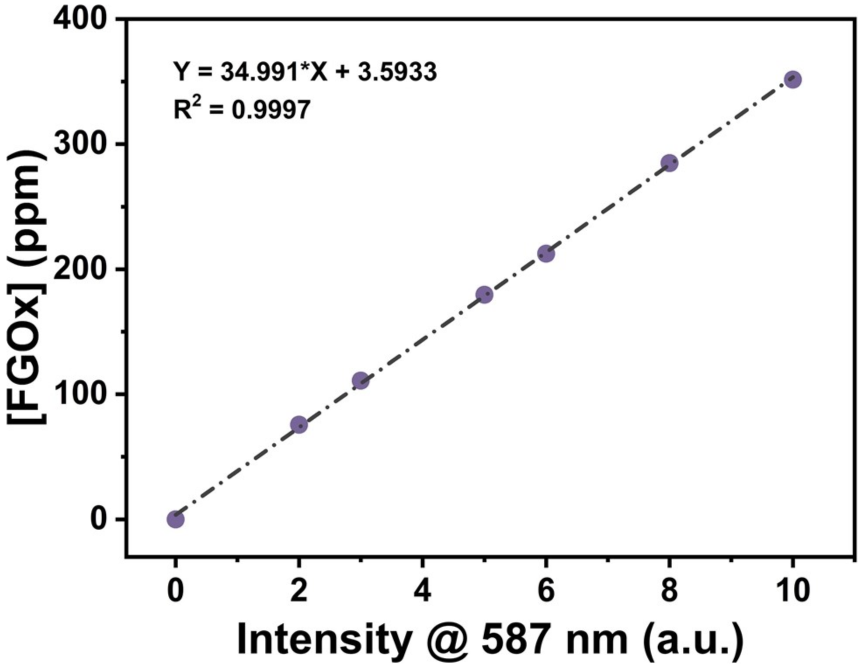

Figure S32. Plot of the standard curve for quantification of GOx by fluorescence.

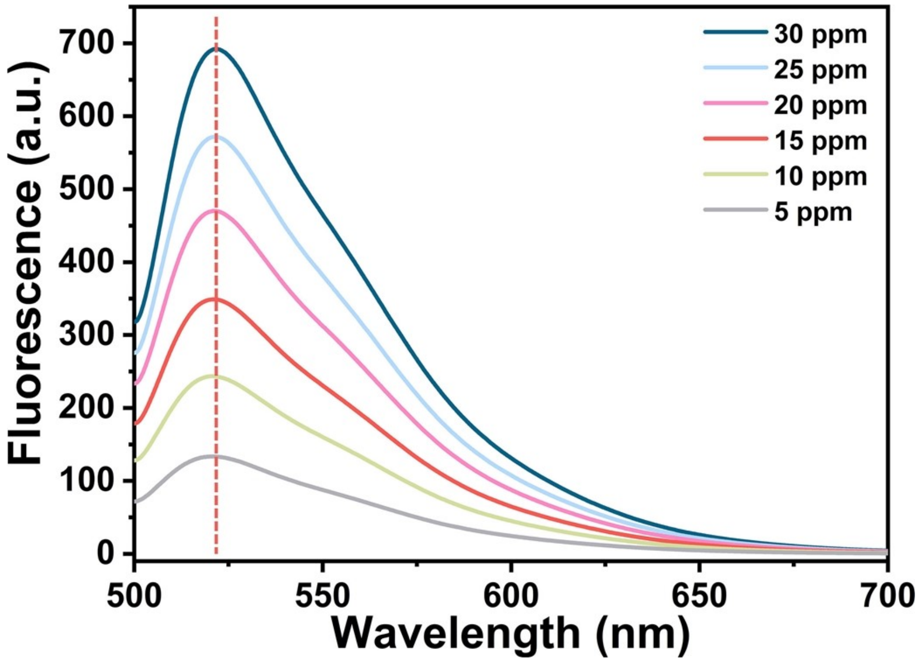

Figure S33. Fluorescence curve of quantitative HRP by fluorescence method.

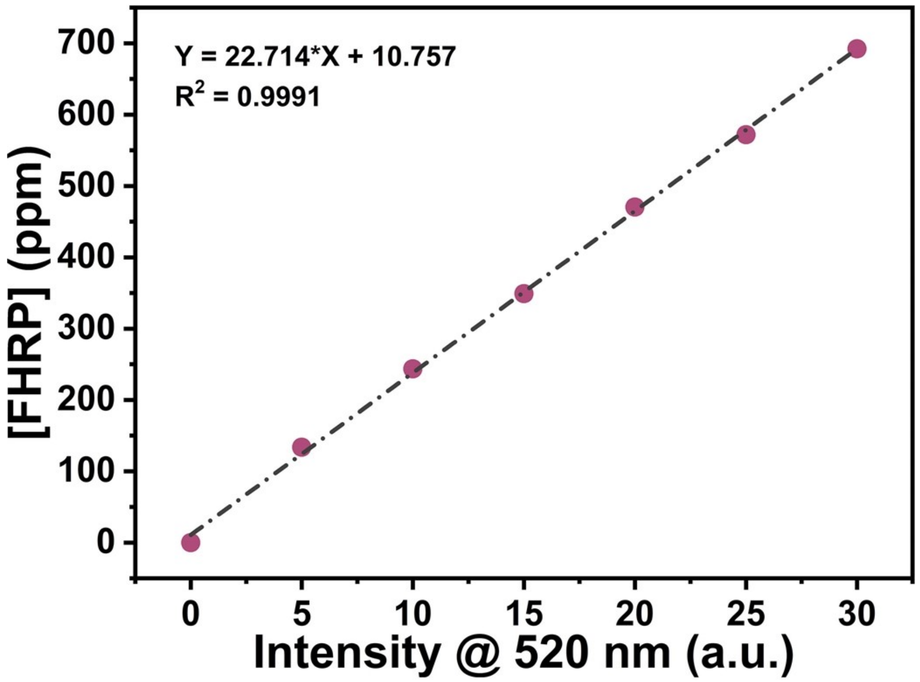

Figure S34. Plot of the standard curve for quantification of HRP by fluorescence.

Table S6. Calculated GOx and HRP loadings in GOx&HRP@HOF-PTBA based on fluorescence tests.

GOx&HRP@HOF-PTBA	Average
**GOx (wt%)**	11.2
**HRP (wt%)**	11.9

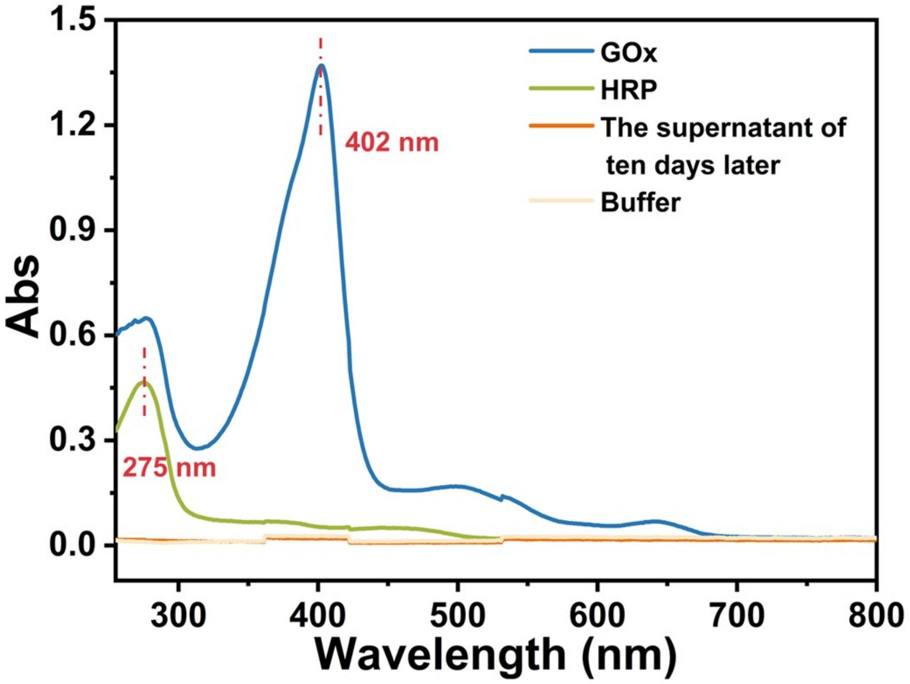

Figure S35. UV-Vis spectra of the collected supernatants after biomimetic mineralization process.

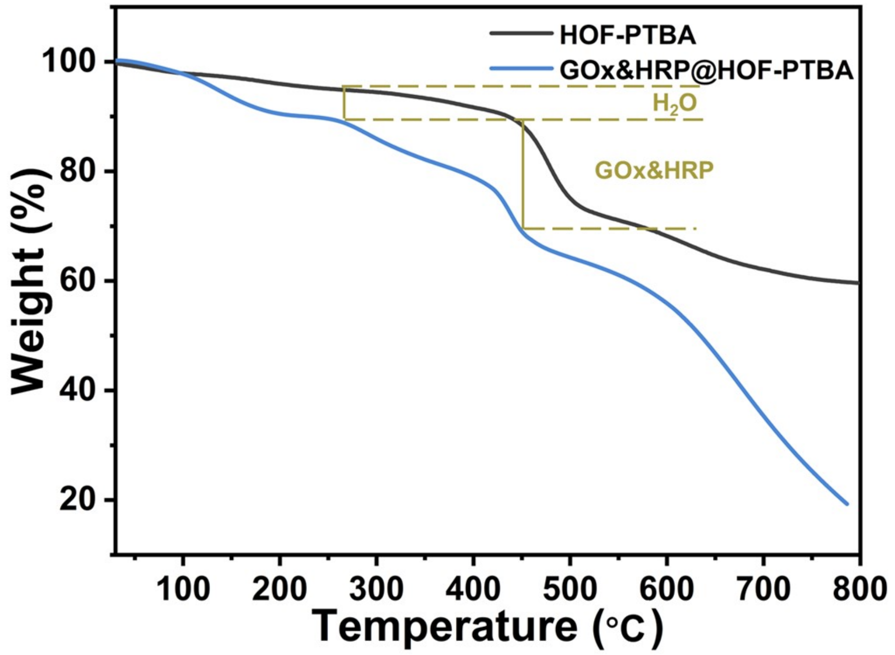

Figure S36. TGA curves of HOF-PTBA and GOx&HRP@HOF-PTBA.

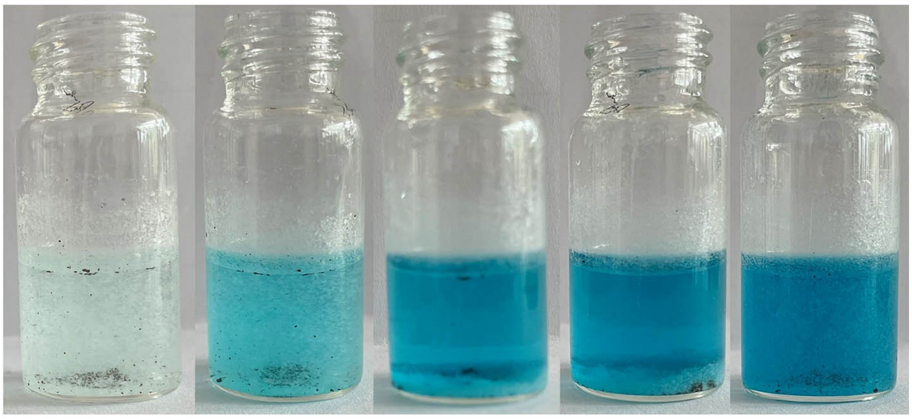

Figure S37. Color of the solution changes to blue (oxTMB) after the cascade catalytic reaction of GOx&HRP@HOF-PTBA.

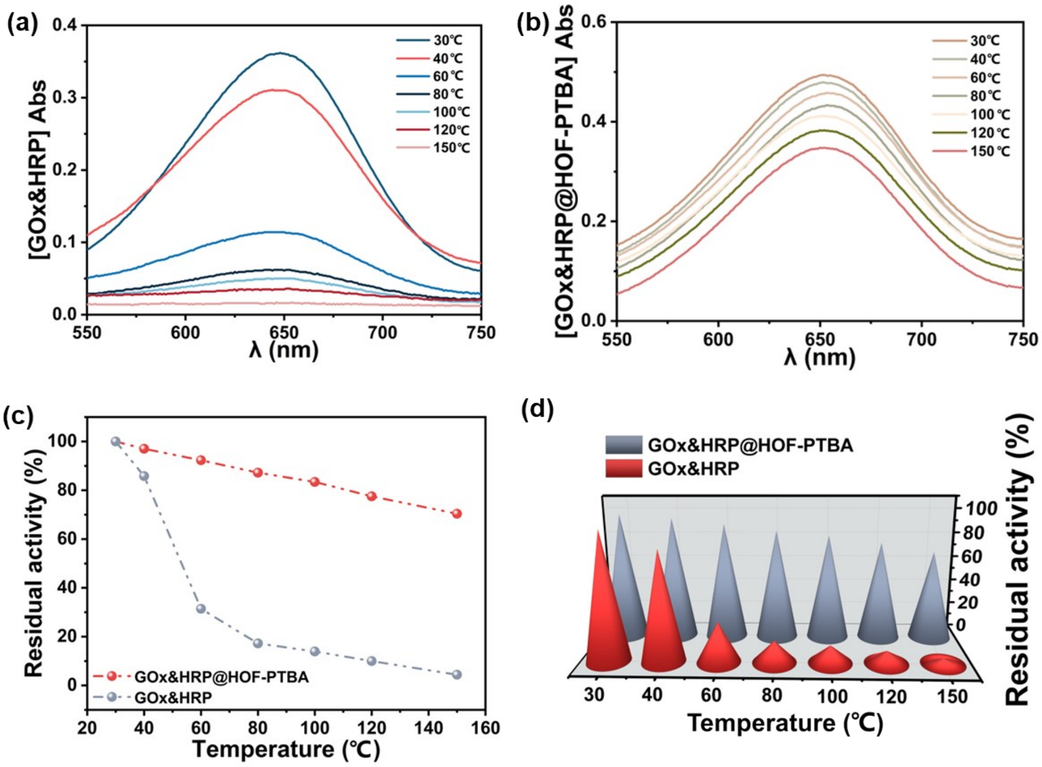

Figure S38. UV-Vis spectra of GOx&HRP (a) and GOx&HRP@HOF-PTBA (b) under different catalytic conditions of various temperatures. Comparison of relative catalytic activity of GOx&HRP and GOx&HRP@HOF-PTBA (c,d). The activity under 30 ºC was normalized as 100%.

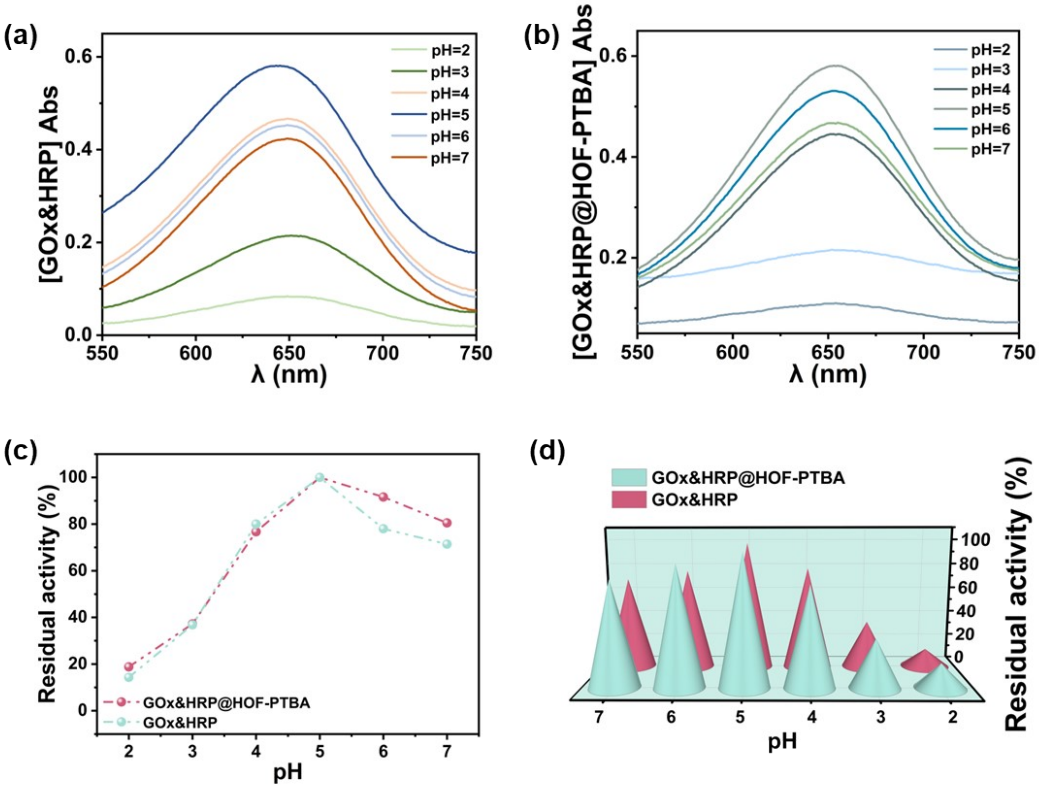

Figure S39. UV-Vis spectra of GOx&HRP (a) and GOx&HRP@HOF-PTBA (b) under different catalytic conditions of various pH buffer solution. Comparison of relative catalytic activity of GOx&HRP and GOx&HRP@HOF-PTBA (c,d). The enzyme activity in pH = 5 was normalized as 100%.

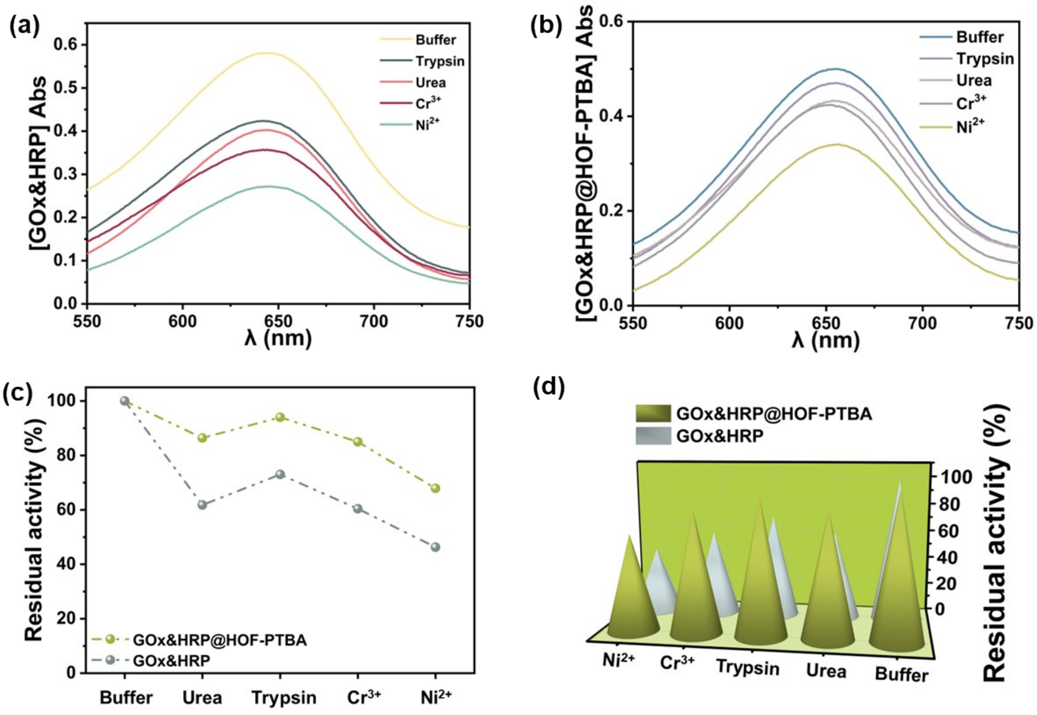

Figure S40. UV-Vis spectra of GOx&HRP (a) and GOx&HRP@HOF-PTBA (b) under different catalytic conditions of various deactivating reagents. Comparison of relative catalytic activity of GOx&HRP and GOx&HRP@HOF-PTBA (c,d). The enzyme activity in buffer solution was normalized as 100%.

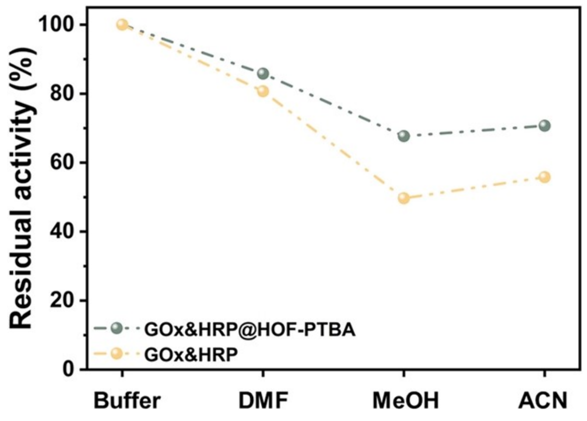

Figure S41. Residual activity of GOx&HRP@HOF-PTBA after treatment by organic solvents.

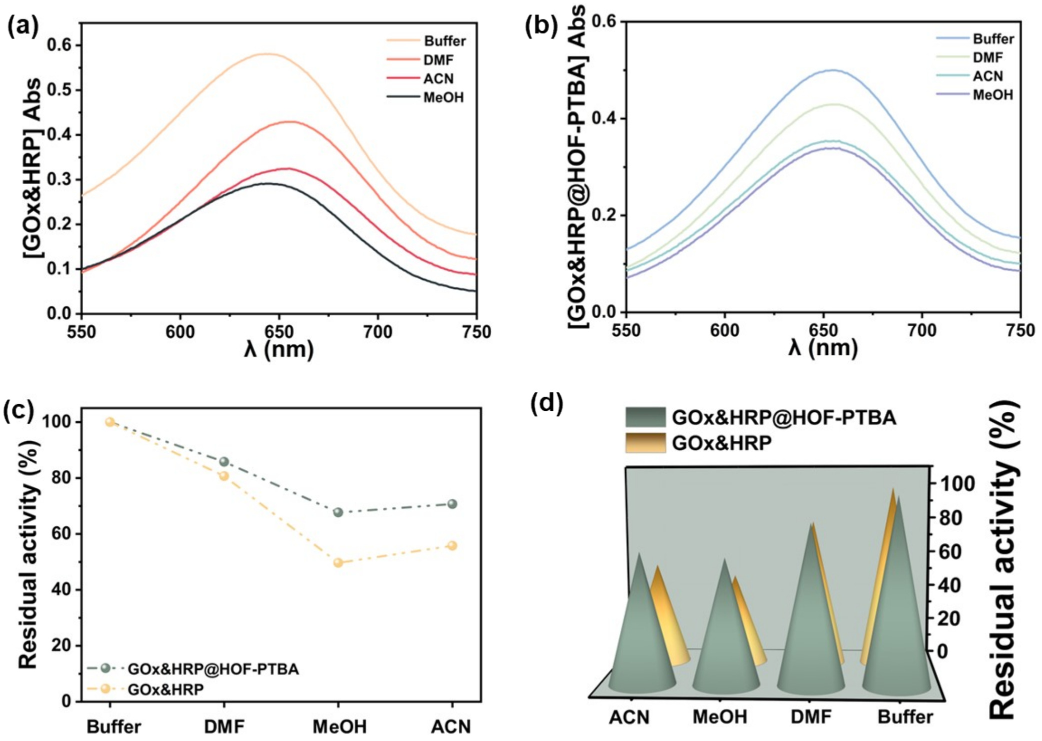

Figure S42. UV-Vis spectra of GOx&HRP (a) and GOx&HRP@HOF-PTBA (b) under different catalytic conditions of various solvents. Comparison of relative catalytic activity of GOx&HRP and GOx&HRP@HOF-PTBA (c,d). The enzyme activity in buffer solution was normalized as 100%.

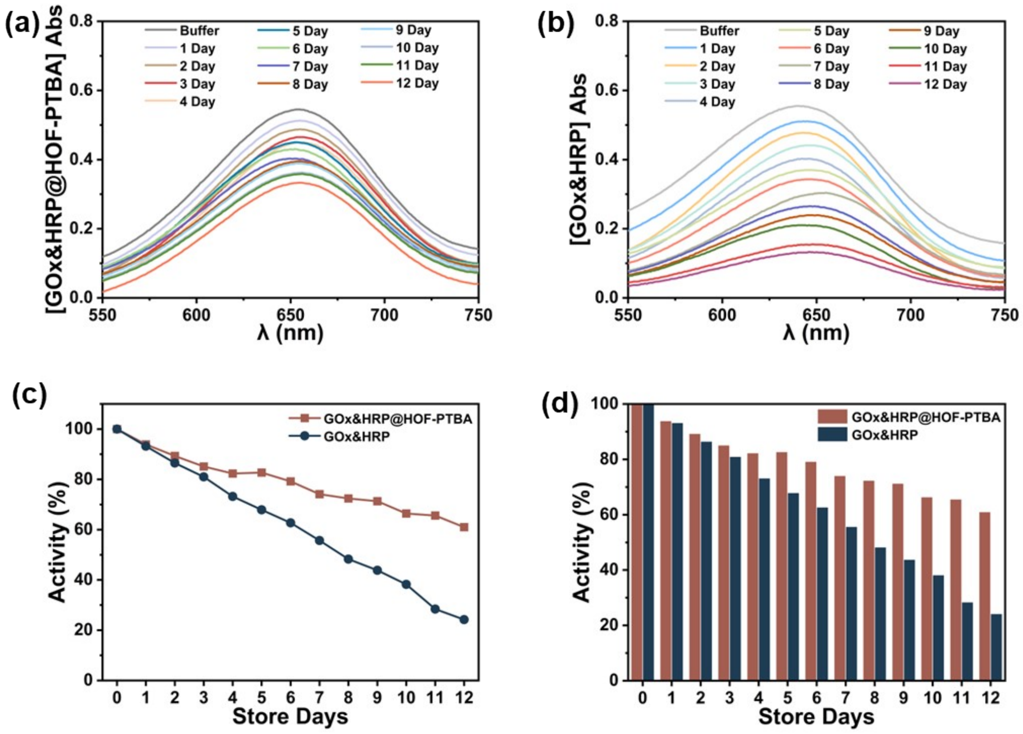

Figure S43. UV-Vis spectra of GOx&HRP (a) and GOx&HRP@HOF-PTBA (b) after different store days. Comparison of relative catalytic activity of GOx&HRP and GOx&HRP@HOF-PTBA (c,d). The enzyme activity in the initial stage was normalized as 100%.

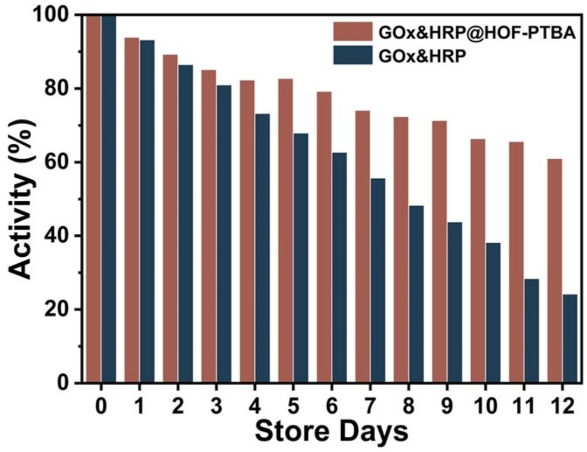

Figure S44. Residual activity of GOx&HRP@HOF-PTBA after keeping at 35 °C for several days.
